# Polyzoa is back: The effect of complete gene sets on the placement of Ectoprocta and Entoprocta

**DOI:** 10.1126/sciadv.abo4400

**Published:** 2022-07-01

**Authors:** Konstantin Khalturin, Natalia Shunatova, Sergei Shchenkov, Yasunori Sasakura, Mayumi Kawamitsu, Noriyuki Satoh

**Affiliations:** 1 Marine Genomics Unit, Okinawa Institute of Science and Technology Graduate University, 1919-1 Tancha, Onna-son, Okinawa 904-0495, Japan.; 2 Department of Invertebrate Zoology, St. Petersburg State University, Saint-Petersburg, Russia.; 3 Shimoda Marine Research Center, University of Tsukuba, Shimoda, Shizuoka 415-0025, Japan.; 4 DNA Sequencing Section, Okinawa Institute of Science and Technology Graduate University, 1919-1 Tancha, Onna-son, Okinawa 904-0495, Japan.

## Abstract

The phylogenomic approach has largely resolved metazoan phylogeny and improved our knowledge of animal evolution based on morphology, paleontology, and embryology. Nevertheless, the placement of two major lophotrochozoan phyla, Entoprocta (Kamptozoa) and Ectoprocta (Bryozoa), remains highly controversial: Originally considered as a single group named Polyzoa (Bryozoa), they were separated on the basis of morphology. So far, each new study of lophotrochozoan evolution has still consistently proposed different phylogenetic positions for these groups. Here, we reinvestigated the placement of Entoprocta and Ectoprocta using highly complete datasets with rigorous contamination removal. Our results from maximum likelihood, Bayesian, and coalescent analyses strongly support the topology in which Entoprocta and Bryozoa form a distinct clade, placed as a sister group to all other lophotrochozoan clades: Annelida, Mollusca, Brachiopoda, Phoronida, and Nemertea. Our study favors the evolutionary scenario where Entoprocta, Cycliophora, and Bryozoa constitute one of the earliest branches among Lophotrochozoa and thus supports the Polyzoa hypothesis.

## Teaser

The earliest lineage among the Lophotrochozoa is identified.

## INTRODUCTION

The phylogenetic relationships between metazoan taxa and the animal evolution pathways have long been key questions for researches. Currently, three main lineages are recognized among bilaterians: Lophotrochozoa, Ecdyzozoa, and Deuterostomia (*1*, *2*, *3*). Although Lophotrochozoa is a well-supported clade, the relationships within it are still unresolved (*1*, *3*–*16*).

**Fig. 1.**
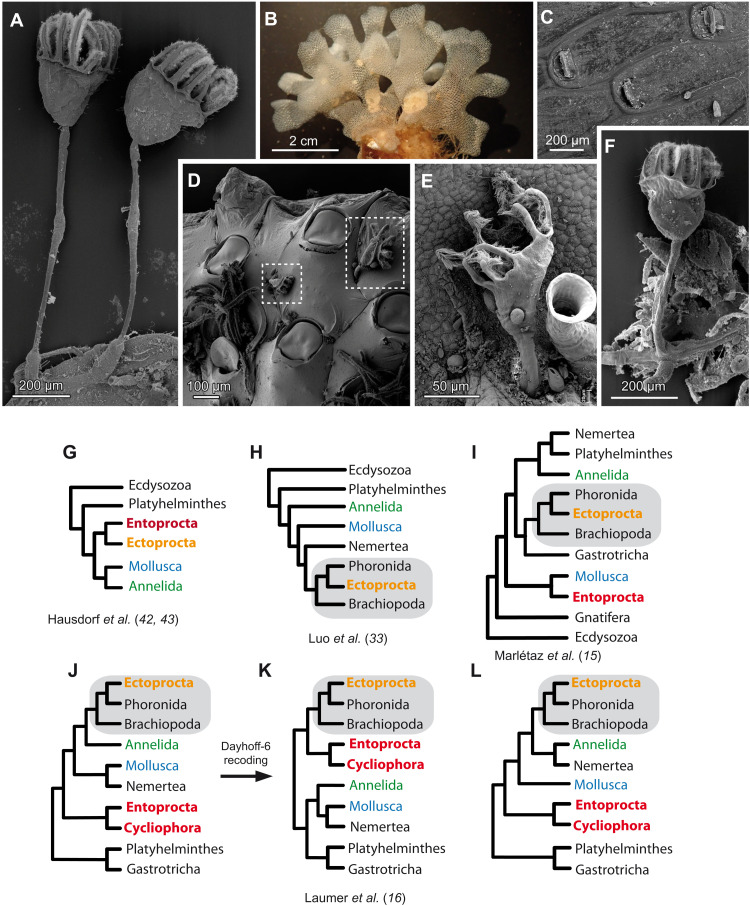
General view and phylogenetic position of Entoprocta and Ectoprocta proposed in previous studies. (**A**) Part of *B. gracilis* colony, scanning electron microscopy (SEM) image. (**B**) General view of *T. membranaceotruncata* colony and (**C**) SEM image of *T. membranaceotruncata* autozooids. (**D**) SEM image of *L. nordgardii* individuals on the surface of a bryozoan colony. (**E**) SEM image of *L. nordgardii* on the surface of algae with *Folliculina* sp. ciliate next to it. (**F**) SEM image of the colony part in *P. cernua*. (**G**) A scheme proposed by Hausdorf *et al.* (*42*, *43*). (**H**) Affinity of Ectoprocta to Phoronida by Luo *et al.* (*33*). (**I**) Separate placement of the taxa by Marlétaz *et al.* (*15*). (**J**) Separate positions of Entoprocta + Cycliophora and Ectoprocta by Laumer *et al.* (*16*). (**K**) Ectoprocta and Entoprocta in one clade after Dayhoff-6 transformation. (**L**) Third version of lophotrochozoan topology by Laumer *et al.* (*16*).

Among Lophotrochozoa, the placement of Bryozoa (=Ectoprocta) and Kamptozoa (=Entoprocta) remains most questionable, and in a historical perspective, the use of different approaches and methods has led to contrasting phylogenies. Both taxa comprise small benthic suspension feeders (Fig. 1, A to F), which were initially assigned to Zoophyta. On the basis of the presence of ciliated tentacles and separated digestive tracts, Thompson (*17*) and Ehrenberg (*18*) distinguished them under the name of Polyzoa or Bryozoa, respectively. Later on, the Bryozoa/Polyzoa group was divided by Nitsche (*19*) and Hatschek (*20*) according to the different location of the anal opening (either inside the tentacle crown in Entoprocta or outside in Ectoprocta), and differences in the general body design (acoelomate in Entoprocta and coelomic in Ectoprocta). Traditionally, according to morphological data, ectoprocts were combined together with phoronids and brachiopods in the group Tentaculata (*20*) or Lophophorata (*21*), whereas entoprocts were proposed to be associated with annelids (*21*, *22*) or mollusks (*23*, *24*). However, Nielsen (*11*, *25*–*28*) claimed Ectoprocta to be very closely related with Entoprocta. After the discovery of Cycliophora, another group of small suspension feeders, Funch and Kristensen (*29*) suggested them to be related to Bryozoa and Entoprocta, and Cavalier-Smith (*30*) resurrected the name Polyzoa for these three phyla. However, this grouping was not widely accepted.

The application of the phylogenomic approach yields contradictory results: Some studies still favor Lophophorata monophyly (*15*, *31*–*35*), while many researches abandon the Lophophorata concept (*1*, *3*, *4*, *14*, *36*–*41*). The placement of the lophotrochozoan groups in different studies is also contradictory (Fig. 1, G to L) and depends on the set of selected markers and taxa sampling in a given analysis (*15*, *16*, *33*, *42*, *43*). During past years, along the transition from small gene sets to large multigenes matrices, many researchers have pointed out that the resolution of phylogenomic analysis is affected by insufficient phylogenetic signal, limitations in taxon and/or gene sampling, or systematic errors and have suggested to apply recoding schemes to decrease data heterogeneity (*14*, *16*, *44*–*49*). However, the question of relationships among Lophotrochozoa proved to be difficult to unambiguously resolve with any of the methods and datasets used so far. Multiple conflicting topologies are certainly unsatisfactory, and some of the likely reasons for this might be purely technical: The usage of unsaturated transcriptomes from Ectoprocta and Entoprocta in the lack of genomic data, possible contaminations due to the tiny size and sessile life style of both groups, and unbalanced taxonomic sampling.

Here, we present a reinvestigation of the phylogenetic position and relationships of Entoprocta and Entoprocta. We attempted to improve the robustness of the phylogeny reconstruction by paying special attention to several aspects: (i) the completeness of the protein set for each species of Ectoprocta and Entoprocta, (ii) screening of protein sets for known sources of potential contamination, and (iii) comparison of topologies based on the marker sets with increasingly strict selection criteria and several recoding schemes. We also examined the topologies recovered by coalescence analysis and the pattern of orthologs distribution among the lophotrochozoan clades.

## RESULTS AND DISCUSSION

### Quality of newly sequenced transcriptomes

**Fig. 2.**
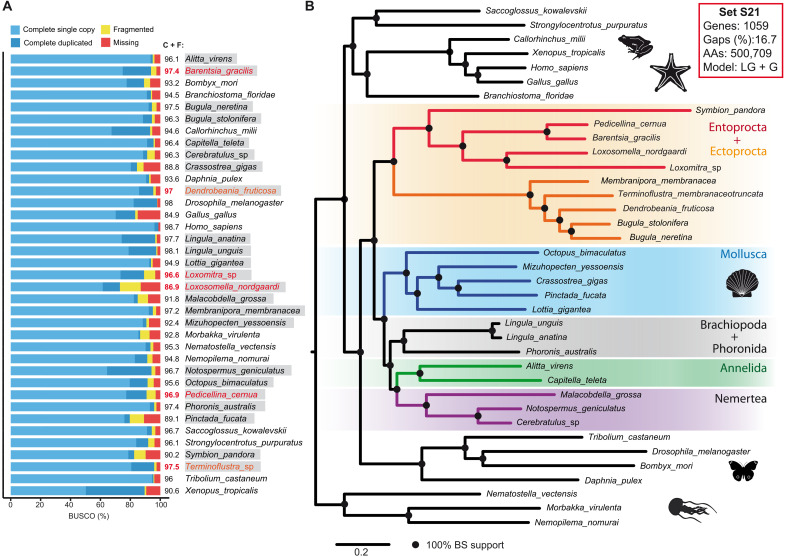
Quality of the datasets used and the ML tree based on 1059 protein markers. (**A**) Quality assessment of the datasets used for phylogeny reconstruction by BUSCO. Completeness (C + F, complete + fragmented) of all newly generated proteins sets, except *Loxosomella*, exceeds 96.6%, which corresponds to the quality range typical for genome projects. Lophotrochozoan species are highlighted with gray background, and six newly sequenced species are shown in red. (**B**) ML tree based on concatenation of 1059 protein markers present in all 37 species. LG + G substitution model, 500,709 distinct alignment patterns, and all branches have maximum BS. AAs, amino acids.

To obtain high-quality reference proteomes, we sequenced transcriptomes of four entoprocts [*Barentsia gracilis* (Sars, 1835), *Loxosomella nordgaardi* Ryland, 1961, *Pedicellina cernua* (Pallas, 1774), and *Loxomitra* sp.)], two ectoprocts [*Terminoflustra membranaceotruncata* (Smitt, 1868) and *Dendrobeania fruticosa* (Packard, 1863)], and applied rigorous two-step decontamination procedure (fig. S1 and tables S1 to S5). Except for *Loxosomella*, all protein sets from the newly sequenced transcriptomes of Ectoprocta and Entoprocta have the sum of Benchmarking Universal Single-Copy Ortholog (BUSCO) values for complete and fragmented sequences above 96% (C + F in Fig. 2A and table S6). In the recent publication (*16*), these values for Ectoprocta and Entoprocta ranged from 40.8 to 88.9 and 18.6 to 61.6, respectively (see table S6). Thus, the completeness of our proteomes by far exceeds that of all Ectoprocta and Entoprocta datasets previously used for phylogenetic reconstructions. In our view, data completeness and quality are crucial to address difficult phylogenetic questions since these eliminate at least some sources of errors, leading to fluctuations in resulting trees (*47*, *50*). The current state of sequencing technology allows generating high-quality transcriptomes (and the proteomes thereof) from any species if enough effort is applied. Thus, there is no more reason to use low-quality datasets with large proportion of missing genes for phylogenomics.

### Large dataset without missing genes

The dataset for our analysis was based on 37 proteomes in total, with five species of Ectoprocta and four species of Ectoprocta (Fig. 2A); details of taxon sampling are given in Materials and Methods. Because of the excellent completeness of the proteomes, we decided not to allow any missing genes in our matrices. Using OrthoFinder with default settings, we identified 1059 orthologs present in all 37 species selected for phylogenetic reconstruction (39,183 proteins in total). After alignment, trimming, and concatenation, the resulting amino acid matrix, designated as the set S21 (see Fig. 2B), contained 500,709 distinct alignment patterns and only 16.7% of gaps and undetermined characters. The maximum likelihood (ML) tree obtained with the set S21 using LG + G model with partitioning is shown in Fig. 2B. All branches have the maximum bootstrap support (BS). As expected, representatives of Deuterostomia and Ecdysozoa form two separate clades, which are sister groups to Lophotrochozoa. The topologies within Cnidaria, Ecdysozoa, and Deuterostomia fully correspond to previous knowledge. Within the Lophotrochozoa, Entoprocta + Entoprocta + *Symbion* form a distinct single clade, which branches out before other lophotrochozoan groups. Within this clade, *Symbion* branches out before all representatives of Entoprocta, supporting thereby the sister relationships between Cycliophora and Entoprocta. ML results also support a group consisting of Phoronida and Brachiopoda as a sister clade to Annelida and Nemertea. This result corresponds well to the previously published studies of spiralian phylogeny based on nonrecoded data where Polyzoa group has been recovered (*3*, *9*, *12*, *51*).

### Information-to-noise ratio

**Fig. 3.**
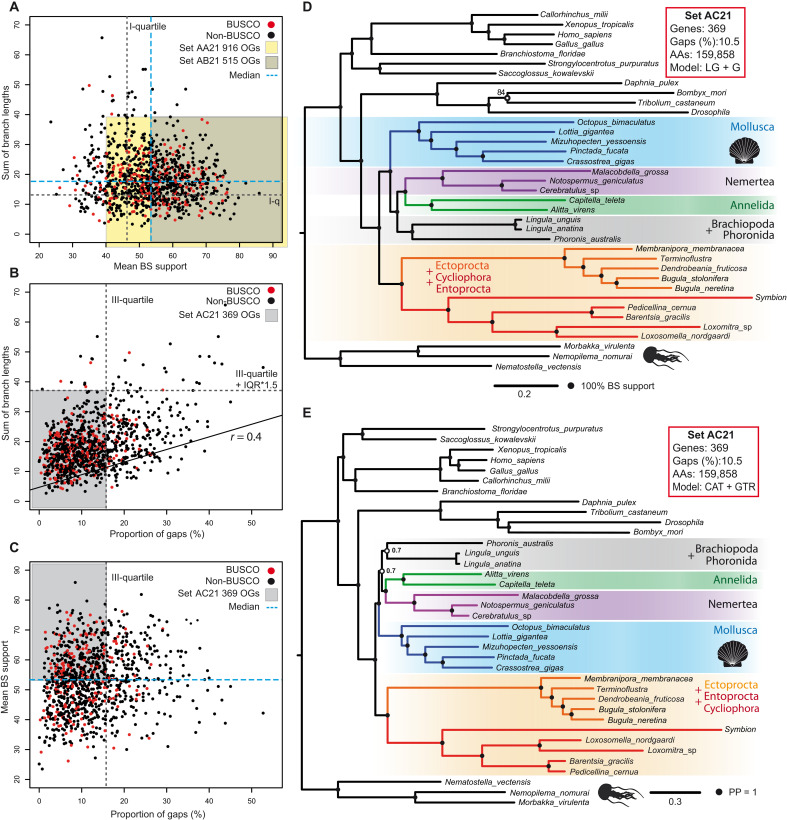
Parameter distributions of the dataset S21 and the phylogenetic trees based on 369 protein markers. (**A** to **C**) Partitioning of S21 set based on (A) the branch lengths, (B) the mean BS, and (C) percentage of gaps in individual gene trees. Red dots denote genes that belong to BUSCO category, and black dots correspond to all remaining proteins. Position of the median values and selected quartile values are shown by dashed lines. IQR, interquartile range. (**D**) ML tree based on concatenation of 369 protein markers (set AC21). LG + G substitution model, 159,858 distinct alignment patterns, and all branches except one marked with open circle have maximum BS. (**E**) BI based on concatenation of 369 protein markers (set AC21). CAT + GTR + G substitution model and all branches except those marked with open circles have maximum support.

On the basis of the information from the individual gene trees, we selected several subsets of genes from the set S21 to increase the information-to-noise ratio of the resulting matrices (Fig. 3, A to C, and fig. S1). This allowed us to test the influence of marker selection and to reduce the matrix to the size optimal for Bayesian inference (BI). Applying increasingly stringent selection criteria for BS, branch length, and alignment gaps, we gradually reduced the number of genes used and constructed three datasets: AA21, AB21, and AC21 (Fig. 3, A to C, and fig. S1). In the datasets AA21 and AB21, the reduction in gene number changes neither topology nor BSs of the resulting ML trees, showing that the markers we used support a single topology, which is stable and not influenced by few outlier genes observed in the dot plots (fig. S2, A and B).

For the AC21 set, the topology of the resulting ML tree within Lophotrochozoa remains identical to the sets S21, AA21, and AB21 (Fig. 2B and fig. S2, A and B) and has maximal support for all branches (Fig. 3D), except for the *Bombyx* + *Tribolium* clade (84%). Discrepancies with larger datasets are observed only within the Ecdysozoa clade, where *Drosophila* swapped its position with *Tribolium* as the earliest branching species. In the Bayesian approach under the CAT + GTR + G model, the AC21 set never reached perfect level of convergence even after >23,000 cycles (maxdiff = 1 and meandiff = 0.028169). The main reason is the uncertainty in relative positions of the Phoronida + Brachiopoda and Annelida + Nemertea clades [posterior probability (PP) = 0.7; Fig. 3E]. The consensus tree, nevertheless, reveals maximum support for Ectoprocta + Entoprocta clade and its placement as the earliest branch within Lophotrochozoa, thus confirming the results of ML analysis (see Fig. 3D). This dataset does not provide a decisive answer about the relative position of Mollusca, (Phoronida + Brachiopoda), and (Annelida + Nemertea) within Lophotrochozoa. Most probably, this set of genes is such that several alternative topologies are supported with equal probabilities, and as a result, the chains never converge. Moreover, the AC21 matrix with ~160,000 positions might be still too large for optimal convergence and mixing in Bayesian approach.

### BUSCO genes

**Fig. 4.**
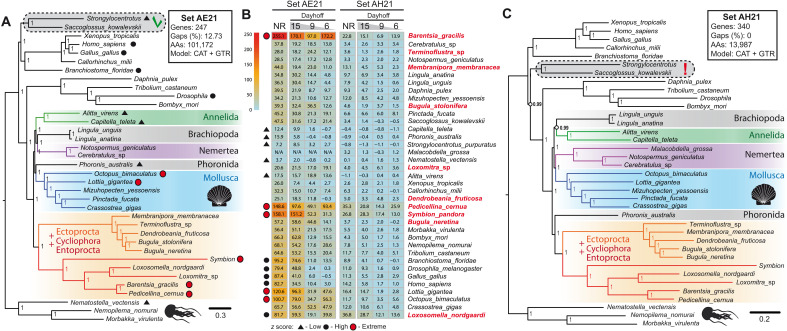
Bayesian analysis and the assessment of compositional heterogeneity. (**A**) BI based on concatenation of 247 protein markers from BUSCO set (AE21). Consensus tree derived from nonrecoded matrix is shown. CAT + GTR + G substitution model, 101,172 distinct alignment positions, 12.7% of gaps and undetermined characters, and all branches have maximum support. (**B**) Results of PPA for nonrecoded (NR) and recoded matrices of the sets AE21 (left) and AH21 (right). The *z* scores are listed for all species and recoding schemes: 6 (Dayhof-6), 9 (Dayhoff-9), and 15 (Dayhoff-15). Species with low, high, and extreme *z* scores are marked with black triangles, black circles, and red circles, respectively. (**C**) BI based on concatenation of 340 protein markers (set AH21). Consensus tree derived from nonrecoded matrix is shown. CAT + GTR + G substitution model, 13,987 distinct alignment positions, 0% of gaps, and all branches except those marked by open circles have maximum support.

To find a more compact but still informative and reliable set of markers, we resorted to the BUSCO genes present in our data. The use of the BUSCO proteins offers several potential advantages: They are well-defined and universal for all Metazoa, are represented by single-copy genes in the majority of metazoan clades, do not have anomalous rates of evolution, and align well among distant taxa (*52*). Of the 978 single-copy orthologs in the BUSCO metazoa_odb9 database, 247 genes were present in all species selected for our phylogenetic analysis; they compose the set AE21 (see fig. S1). On the basis of the set AE21, we reconstructed the phylogeny using Bayesian (Fig. 4A) and ML (fig. S3A) approaches. In the Bayesian analysis, under CAT + GTR + G model, we again observe a topology in which the clade consisting of Cycliophoira + Entoprocta + Ectoprocta is the earliest branching group among Lophotrochozoa (Fig. 4A). However, the Brachiopoda + Phoronida clade splits into two groups, with Brachiopoda placed together with Nemertea, and Phoronida branches off immediately before Mollusca representatives. Thus, the topology in the Bayesian analysis contradicts the ML results recovered from the same dataset (fig. S3A), where Phoronida and Brachiopoda are placed as sister groups, which is a conventional topology recovered in the previous investigations of spiralian phylogeny (*15*, *16*, *33*, *51*).

### Assessing the compositional heterogeneity of data

Recently, it has been suggested that composition heterogeneity is one of the factors compromising the correct phylogenetic placement of Ectoprocta and Entoprocta (*14*, *16*). For the set AE21, posterior predictive analysis (PPA) shows that even the CAT + GTR + G model, which explicitly accounts for site-specific amino acid preferences, poorly accounts for compositional variation in the dataset except for that in *Nematostella* (Fig. 4B, left). Among Entoprocta, the *z* score values of PPA results range from 20 in *Loxomitra* to 255 in *Barentsia*. Among Ectoprocta, the variations are not so strong: from 25 in *Dendrobaenia* to 57 in *Bugula*. The compositional heterogeneity of *Symbion* also approaches the upper range with a *z* score of 158, close to that in *Pedicellina*. It is worth noting that high levels of compositional heterogeneity are by no means limited to Ectoprocta and Entoprocta. For example, in human and chicken, PPA score exceeds 80, being close to that in *Drosophila*, whereas in *Lottia* and in *Octopus*, it exceeds 100. However, this feature of the dataset does not seem to compromise the proper topology within Deuterostomia, Ecdysozoa, or Mollusca. We also do not observe any grouping of the five species with the highest level of compositional heterogeneity (*Barentsia*, *Symbion*, *Pedicellina*, *Lottia*, and *Octopus*, marked by filled red circles in Fig. 4A). Thus, it seems that despite nonperfect model fit, true phylogenetic signal overweighs the noise created by the uneven frequencies of amino acid substitutions observed in several species.

To find out whether data transformation approaches can help reduce the compositional heterogeneity, we tested three different data recoding strategies: the standard Dayhoff-6 transformation scheme into six classes (*53*) and recoding into 9 (Dayhoff-9) and 15 (Dayhoff-15) categories based on PAM250 transition matrix (see Materials and Methods for details). The latter two schemes were originally proposed and validated on simulated datasets in (*54*). We decided to apply them to our data as they are less strict than Dayhoff-6 scheme and, therefore, may better preserve phylogenetic signal. In general, analysis having the lower scores in PPA is considered to be the most adequate. As shown in Table 1, all three data recoding strategies effectively decreases scores for all five PPA statistics. Negative *z* scores indicate that amino acid diversity is overestimated by the model after Dayhoff-6 and Dayhoff-9 recoding, whereas the diversity is largely underestimated using the nonrecoded matrix. However, different PPA statistics show that there is no uniform improvement of adequacy from nonrecoded to recoded data (see Table 1). For example, the best scores for PPA-DIV and PPA-MAX are observed in Dayhoff-9, while PPA-CONV and PPA-VAR have better scores in the nonrecoded scheme. Hence, in this example, by applying recording, we effectively reduce lineage-specific compositional heterogeneity, but the model becomes less efficient in describing across-site compositional heterogeneity. Together, it is actually difficult to decide, on the basis of PPA analysis alone, which strategy of data transformation will result in the most reliable phylogenetic reconstruction.

**Table 1.** Results of PPA for the sets AE21 and AH21. For each set, the results from nonrecoded and Dayhoff-6, Dayhoff-9, and Dayhoff-15 matrices are shown for comparison. The best fitting parameter in each group is highlighted in bold. In all cases CAT + GTR + G model was used. None of the recoding schemes has the absolute advantage in all five parameters.

**Matrix/recoding scheme**	**Model**	**Site-specific amino** **acid preferences**	**Across-taxa** **compositional heterogeneity**	**Empirical** **convergence** **probability**	**Across-site** **compositional** **heterogeneity**	**Mean squared** **heterogeneity** **across taxa**
		**PPA-DIV**	**PPA-MAX**	**PPA-CONV**	**PPA-VAR**	**PPA-MEAN**
Set AE21:						
None	CAT + GTR	6.50338	138.335	**8.1909**	**8.8697**	313.752
Dayhoff-6	CAT + GTR	−1.7959	82.4557	10.9337	11.0215	106.943
Dayhoff-9	CAT + GTR	**−0.6288**	**45.4654**	11.6434	12.1904	**101.149**
Dayhoff-15	CAT + GTR	4.3768	98.1346	14.1153	13.8091	226.055
Set AH21:						
None	CAT + GTR	4.9079	39.0857	**2.83906**	**2.78946**	48.7904
Dayhoff-6	CAT + GTR	**0.1449**	18.7388	5.4639	5.8251	**19.8551**
Dayhoff-9	CAT + GTR	0.8086	**18.5237**	6.0536	6.0197	20.1687
Dayhoff-15	CAT + GTR	3.3539	33.3942	5.7612	4.9709	34.7670

The phylogenetic trees obtained in the Dayhoff-6 and Dayhoff-9 recoded analyses of the set AE21 have a markedly different topology from their nonrecoded counterpart (fig. S3, A to C). Namely, Lophophorata (Brachiopoda + Phoronida + Ectoprocta) is the earliest branch among Lophotrochozoa, Mollusca is a sister group to Cycliophora + Entoprocta, and Annelida + Nemertea constitutes the third group (fig. S3, B and C). This grouping of taxa perfectly recapitulates the phylogeny previously published by Marlétaz *et al*. (*15*), where CAT + GTR model was combined with Dayhoff-6 recoding (Fig. 1I). The only difference is the position of the Mollusca + Entoprocta clade, which, in (*15*), is the earliest branching clade within Lophotrochozoa. Analyses of the recoded matrices by Laumer *et al*. (*16*) also favor the presence of Lophophorata (see Fig. 1, J to L), but the Cycliophora + Ectoprocta clade either occupies the most basal position or groups together with Lophophorata. Although the Dayhoff-6 recorded data score well in PPA, a serious concern is that the resulting tree violates the monophyly of Deuterostomia, because Echinodermata and Hemichordata do not form a sister clade with Chordata (fig. S3B). The Dayhoff-9 recoding scheme restores the conventional topology within Deuterostomia with all branches having maximum support (fig. S3C). In the case of Dayhoff-15 recording, a third alternative topology appears (fig. S3D); in general, it resembles the results of Dayhoff-6 recoded analysis by Laumer *et al*. (*16*), where Lophophorata and Cycliophora + Entoprocta are grouped together (see Fig. 1K). In our Dayhoff-15 analysis, however, the relationship among these four clades is not resolved (fig. S3D). This observation calls into question the usefulness of recoded AE21 set. The contradicting topologies resulted from the analysis of the same matrix with different recoding schemes are problematic in themselves; moreover, the main question thereby remains unanswered. Formally, on the basis of theoretical assumptions, the Dayhoff-6 recording, which gives lower scores in PPA compared to the nonrecoded data, should give us a more reliable reconstruction of evolutionary trajectories. However, there are reasons to doubt this approach if we observe topologies that strongly contradict previous knowledge about the phylogenetic relations within outgroups. In this case, the simplest interpretation is that the dataset had been artificially brought to such an extreme level of homogeneity that the genuine phylogenetic signals are already lost.

### Removing the main source of compositional heterogeneity

Our initial dataset S21 with 1059 genes (520,348 residues, 16.7% missing data) is larger and more complete than any dataset previously used to examine the position of Ectoprocta and Entoprocta. To test whether the complete removal of columns containing even a single gap among 37 species would improve the fit of the data to CAT + GTR + G model, we constructed the set AH21, represented by 13,987 informative positions from 340 genes and 0% of missing data. According to PPA, in its nonrecoded form, AH21 set outperforms Dayhoff-6 and Dayhoff-9 recorded analyses of set AH21 in all statistics except PPA-DIV scores (Table 1). However, it is a matter of a debate what is better: a slight underestimation of diversity in AH21 (PPA-DIV = 4.9079) or an overestimation in AE21 (PPA-DIV = −1.7959 and −0.6288). Low *z* scores observed in AH21 indicate that a considerable proportion of compositional heterogeneity observed in Entoprocta, Ectoprocta, and other taxa most probably comes from the alignment gaps.

In Bayesian analysis, the set AH21 converges well, which is not unexpected for a matrix of small size (maxdiff = 0.0440107 and meandiff = 0.00118931). Under CAT + GTR model with a nonrecoded matrix, we recover the grouping of Phoronida as a sister clade to Ectoprocta + Entoprocta (Fig. 4C). This topology resembles the results of Laumer *et al.* (*16*) under the Dayhoff-6 recording (Fig. 1L) but without Brachiopoda, which, in our case, groups together with Annelida. One concerning observation is once again the lack of monophyly of Deuterostomia, which might indicate signal degradation. In the Dayhoff-15 recoding analysis, we recover identical topology to nonrecoded matrix, while the Dayhoff-9 and Dayhoff-6 recoding demonstrates clear signal erosion since several branches were not resolved (fig. S3, B to D). Together, the sister relations of Entoprocta and Ectoprocta and their placement as the earliest branching group of Lophotrochozoa are the most frequently occurring topology throughout our analyses and datasets.

### Looking for additional hints

**Fig. 5.**
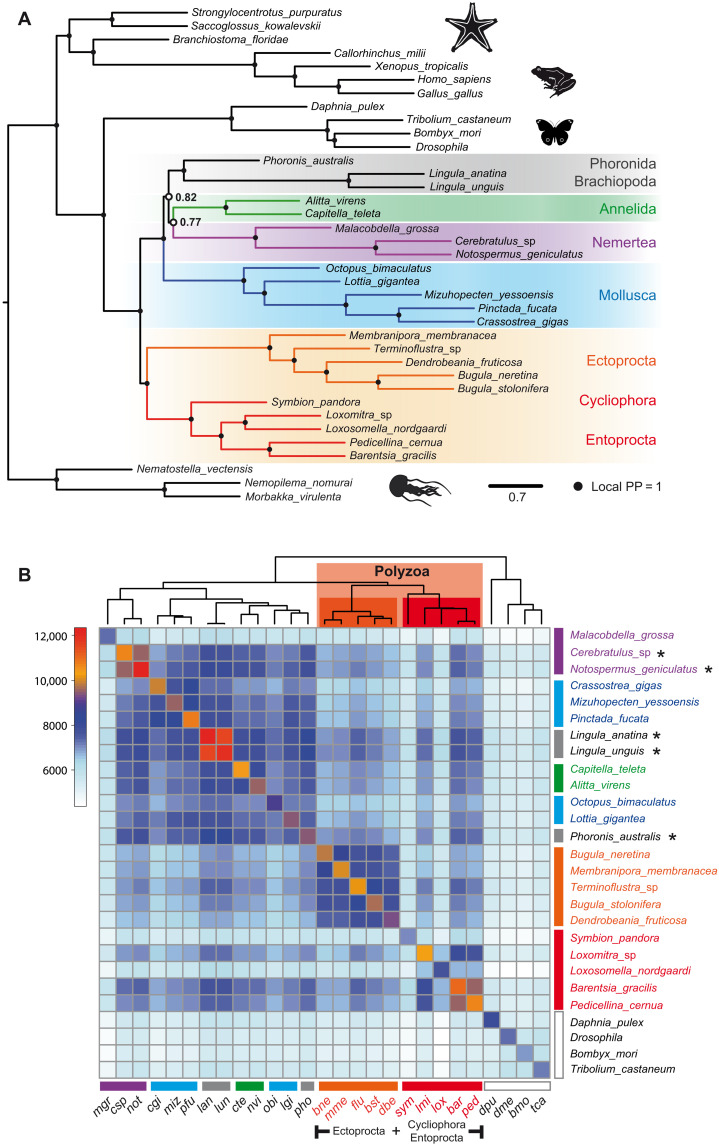
Coalescence analysis and the distribution of shared orthologs among lophotrochozoan lineages. (**A**) The consensus tree of coalescence analysis based on 1036 orthologs present in all 37 species. Branches with a BS of ≤10% were collapsed in individual gene trees before consensus tree calculation. All nodes except those marked with open circles have a local PP of ~1. (**B**) The heatmap represents the number of OGs that are shared among the representatives of Lophotrochozoa and Ecdysozoa used as outgroup. Number of shared OGs is coded with a color gradient bar shown on the left side of the figure. Species were clustered on the basis of the similarities of their gene sets. Result of the clustering is represented by a cladogram on the top of the heatmap. Representatives of Ectoprocta, Entoprocta, and *Symbion* (Cycliophora) form a distinct clade. This group of taxa in marked collectively with orange and red polygons and with “Polyzoa” tag. Asterisks mark the species with the highest number of shared OGs to the representatives of Ectoprocta and Entoprocta.

To obtain additional hints about the placement of Ectoprocta and Entoprocta, we applied coalescent analysis to our data. For the orthologs present in S21 set, 1036 gene trees were constructed by RAxML under LG + G model (referred to as setS21′ in fig. S1). For each gene tree, all branches with BS below 10% were contracted, and the coalescent tree was reconstructed using ASTRAL-III (*55*). As a result, Ectoprocta and Entoprocta form a clade that branched prior to the divergence of all other Lophotrochozoan lineages (Fig. 5A). Topologies within all outgroups are reasonable, and local posterior probabilities are maximal for all nodes, except for those between [Phoronida + Brachiopoda] and [Annelida + Nemertea] (PP ~ 0.82), as well as between Annelida and Nemertea (PP ~ 0.77). Coalescent trees obtained with the sets AB21 and AC21 demonstrate identical topology with respect to the placement of Ectoprocta + Entoprocta as the earliest clade of Lophotrochozoa (fig. S5, A and B). However, there are interesting fluctuations in the local PP support for other clades. For the set AB21, PP value for the Annelida + Nemertea clade increases from 0.77 to 0.98, while support for the Phoronida + Brachiopoda clade as a sister group of the Annelida + Nemertea clade drops from 0.82 to 0.62. This observation implies the presence of genes with contradicting evolutionary signals and indicates that the representatives of Phoronida, Brachiophda, Nemertea, and Annelida are major sources of topological instability in our reconstructions. Expectedly, the resolution of the coalescent approach drops as gene number decreases, and several taxon rearrangements are observed in set AC21, albeit with low support (fig. S5B). At the same time, even in the set AC21, the relationships of species within outgroups remain stable and reasonable, further emphasizing the reliability of genome-derived protein sets.

As an additional source of evidence, we checked the distribution of shared orthologs among the lophotrochozoan lineages (Fig. 5B). Protein sets derived from transcriptomes are certainly not the best source for these comparisons, but given the excellent completeness of our dataset (see Fig. 2A), it was worth a try. The main concern was the not yet saturated datasets for *Loxosomella* (Ectoprocta), *Symbion* (Cycliophora), and *Malacobdella* (Nemertea), where the total number of proteins is small and which may be attracted to each other in cluster analysis. Proteomes of 37 species were clustered by OrthoFinder, and the number of shared orthologous groups (OGs) was calculated for all species combinations (see Materials and Methods for details). The resulting matrix (table S8) was clustered to bring together the species with the most similar gene sets. For the subset of 27 species (complete matrix is shown in fig. S5C), four distinct clusters are evident: Ecdysozoa, Cycliophora + Ectoprocta, Entoprocta, and remaining Lophotrochozoa (Fig. 5B). Contrary to our initial expectations, species with unsaturated proteomes correctly grouped with their respective clades, similar to that in ML and Bayesian analyses (*Symbion* clusters next to Entoprocta). Thus, differences in the absolute number of genes do not obscure the true phylogenetic relationships. The clustering results are not without problems; mollusks are split into two groups by intervening brachiopods and annelids, and *Phoronis* is grouped together with *Lottia* and *Octopus*. Moreover, the complete matrix with 37 species violates the monophyly of Deuterostomia by placing Hemichordates, Cephalochordates, and sea urchin inside Lophotrochozoa (between Brachiopods and Annelids). However, despite several examples of obviously incorrect grouping, this heatmap allows several interesting observations. For example, it is clear that all the species of Entoprocta, except *Loxosomella* (low total number of proteins), have the largest number of OGs in common with *Terminoflustra*, *Phoronis*, and both species of *Lingula* (Brachiopoda). This confirms and explains the affinity of these groups toward each other in all sorts of phylogenetic analyses done here and by others researches previously. Overall, both the coalescent analysis and the comparison of gene sets further confirm the similarities between Ectoprocta and Entoprocta, as well as their probable location as the earliest branching group within Lophotrochozoa.

### Polyzoa is back

Our data support the presence of a clade consisting of Entoprocta, Ectoprocta, and Cycliophora, which branches out before other lophotrochozoan groups. The presence of this clade was found in other studies (*9*, *14*, *51*), and following the idea of Cavalier-Smith (*30*), Hejnol *et al*. (*9*) named this clade Polyzoa. Among other reasons, the variability of topologies reported in the past year depends on the taxon sampling. Thus, Giribet and Edgecombe (*56*) mentioned that the inclusion of Cycliophora in the analyses changes the topology: Some phylogenomic analyses recover a group of Bryozoa + Entoprocta (*12*, *42*, *43*), but this clade often disappears when Cycliophora is added (*9*, *14*, *16*, *32*). A close relationship between ectoprocts, bryozoans, and cycliophorans was originally suggested by Funch and Kristensen (*29*) after the first description of a cycliophoran, *Symbion pandora*. These authors pointed out that cycliophorans, entoprocts, and bryozoans have some similarities in the development of feeding structures and asexual budding. Cycliophorans show more morphological similarities with entoprocts, e.g., in the larval structure (*57*). However, the structure of food-gathering apparatus in these groups differs.

Contrary to the idea of combining Bryozoa and Entoprocta, Hyman (*21*) suggested the concept of Lophophorata, and the Lophophorata monophyly has received some support in recent phylogenomic studies (*14*–*16*, *32*, *35*, *51*). Nevertheless, the morphological similarities of phoronids, brachiopods, and bryozoans are not as convincing as we used to think, and morphologically based conclusions about the relationships of these groups are also contradictory. The core of the Lophophorata concept of Hyman (*21*) lies in the presence of the lophophore and the nature and arrangement of the body cavities, which are coelomic and tripartite. These traits are seen only in phoronids, whereas brachiopods have more complex partitioning of the coelom (*58*–*61*). As for bryozoans, a recent study demonstrated that their body cavity has three main designs: undivided in phylactolaemates, partly separated in two compartments in gymnolaemates, and a combination of two separated coelomic compartments accompanied with a modified primary body cavity in cyclostomes (*62*). Moreover, many gymnolaemates display an acoelomate condition of the main body cavity (*62*, *63*). These facts contradict the core of the Lophophorata concept and suggest that other characters present in bryozoans, phoronids, and brachiopods, such as lophophore and U-shaped gut, could be considered as the result of convergence. The homology of the lophophore in phoronids, brachiopods, and bryozoans was doubted before (*1*). However, some recent studies on the lophophore innervation in phoronids suggest the possibility of homologization of this structure in phoronids, brachiopods, and bryozoans (*64*, *65*). Is Polyzoa an artificial clade? At the current state of knowledge, no more than Lophophorata, to which we are just accustomed. In both cases, the phyla composing each clade share minimum morphological and developmental similarities, and further phylogenomic and morphological studies are needed.

### General remarks

Multigene matrices with a considerable proportion of missing data are routinely used in phylogenomics. This approach is considered reliable on the basis of the assumption that the missing genes are evenly scattered through the data matrix (*47*, *50*). However, the distribution of gaps is not always ideal, and the reliability of the results depends on the genetic differences between the compared species. The greater the proportion of gaps in a matrix and the smaller the genetic distances among the species, the higher the likelihood of potential errors. Since Ectoprocta and Entoprocta have often been among the species with the most missing data, their placement has likely suffered the most.

The problem of missing data has an interesting parallel in the context of current sequencing technologies. No one would dare to build a gene tree based on raw NanoPore or PacBio reads (with up to 15 to 20% of randomly distributed errors or, in other words, “missing” data), but conceptually, a similar thing is routinely done when the multigene matrices with large proportion of gaps are used in phylogenomics. It is surely possible to build a gene tree based on “noisy” single-molecule data, but how reliable will it be? The balance between the sequence divergence and the amount of missing data is decisive for the result. A prerequisite for success is that the differences between sequences must be much higher than the error rate (i.e., random noise or proportion of undetermined states). In this case, the true signal will overweight the “noise” introduced by random errors, and a reasonable tree might be obtained. However, if the original sequences are already very similar, then the noise (errors and Ns) will obscure their true relationships resulting in a tree with unresolved branches and low BSs. The internal branches leading to lophotrochozoan clades are short, and the genetic distances among all these groups are almost identical. It is the low level of sequence differences among lophotrochozoan clades that makes this animal group especially difficult for phylogenetic reconstruction and hampers the placement of Entoprocta and Entoprocta. Thus, to reconstruct evolutionary history of Lophotrochozoa, more genes are needed and protein sets must be as complete as possible. These measures increase signal-to-noise ratio, which is crucially important for this group.

The major improvements of our analysis compared to previous studies are that (i) we used the species of Entoprocta and Ectoprocta with their transcriptomes sequenced to saturation level (except in *Loxosomella*), (ii) missing genes were not allowed in any taxa analyzed, and (iii) strict contamination screening was applied. Together, all of these measures resulted in a data matrix whose size and completeness had not been achieved in any previous study. Thus, on the basis of current datasets and phylogeny reconstruction technology, we obtained the most probable scenario of evolutionary relations between Entoprocta and Ectoprocta, as well as their most probable placement among other lophotrochozoan taxa.

Although our results do not provide a solution to the general phylogeny of Lophotrochozoa, they identify weaknesses in current knowledge and provide a better understanding of what steps need to be taken in the future. We believe that at least two to three high-quality genomes for the representatives of Nemertea, Phoronida, Entoprocta, and Ectoprocta are still needed to improve reliability of phylogenetic reconstructions. At least one additional genome from a slow evolving representative of Annelida would be also a great help, since *Helobdella robusta* is rather derived species with elevated rate of evolution. We also anticipate that the availability of additional genomic resources and comparative approach will allow better investigation of evolutionary trajectories within Lophotrochozoa. Overlaps of gene sets, gene order within genomes, lineage-specific genes, and transposable elements could reveal much more about lophotrochozoan evolution than currently available data sources allow. Thus, the search for the true evolutionary history of Lophotrochozoa has not been completed yet.

Obviously, the most important part of phylogenetic reconstruction is a reliable dataset. We believe that it is important to establish a rigorous quality standard for input protein sets in phylogenomics. There is no universal measure, but BUSCO (*52*) has become the de facto quality standard in the genomics field and can serve as a good starting point to determine the validity of a given protein set for phylogenetic reconstruction. Without such an agreement on the quality of the input data, the resulting phylogenies will continue to contradict each other in the future.

## MATERIALS AND METHODS

A flow chart of the analysis, from sample collection to the resulting data matrices, is shown in fig. S1. The protein sets and data matrices in nonrecoded and recoded form are available as EctoEnto_2021.tar.gz on the web site of the Marine Genomics Unit of Okinawa Institute of Science and Technology (http://compagen.unit.oist.jp/aurelia/datasets.html) with login “guest” and password “welcome.”

### Sampling

Three species of Entoprocta, *B. gracilis* (Fig. 1D), *L. nordgaardi* (Fig. 1, D and E), *P. cernua* (Fig. 1F), and two bryozoans, *T. membranaceotruncata* (Fig. 1, B and C) and *D. fruticosa*, were collected in the vicinity of Educational and Research Center “Belomorskaya” of the St. Petersburg State University in Kandalaksha Bay, White Sea, Russia (table S1). Specimens of *Loxomitra* sp. were obtained from a culturing tank at Shimoda Marine Research Center, Shizuoka, Japan (table S1). Our sampling set included colonial (*B. gracilis* and *P. cernua*) and solitary (*L. nordgaardi* and *Loxomitra* sp.) entoprocts. Small pieces of red algae inhabited by entoprocts were kept in the laboratory at +4°C in sterile-filtered seawater for at least 24 hours to reduce contamination with ingested food. Animals were anesthetized with a mixture of isotonic solution of magnesium chloride and filtered seawater (ratio, 1:1). To prevent contaminations with epibionts, only the upper parts of individual animals that did not contact the substratum were collected using tweezers. In *Loxosomella* and *Loxomitra*, the most basal foot part of each individual was removed. In case of *Terminoflustra* and *Dendrobeania*, individual colonies were collected by scuba diving. Bryozoan colonies were also kept in sterile-filtered seawater for at least 24 hours at +4°C to reduce contamination with food and were thoroughly cleaned with brush to remove detritus and motile animals. We selected only those pieces of colonies that lacked visible epibionts under a stereomicroscope. After dissection specimens were homogenized in 700 μl of TRIzol reagent and kept frozen at −80°C until RNA extraction.

### RNA preparation and sequencing

Total RNA was extracted by combination of standard TRIzol protocol and RNAeasy micro kit (QIAGEN). NanoDrop and Bioanalyzer were used for RNA quantification and quality control. Sequencing libraries were prepared with TruSeq Stranded RNA kit (Illumina) and sequenced on MiSeq, HiSeq 2500, and NovaSeq 6000 instruments (Illumina). Description of each sample and the corresponding National Center for Biotechnology Information (NCBI) BioProject identifiers are given in table S1.

### Transcriptome assembly and contamination screening

Raw sequencing reads were trimmed and quality filtered with Trimmomatic v0.36 (*66*). Transcriptomes were assembled de novo with Trinity v2.3.2 (*67*) using default parameters. Redundant transcripts were removed by applying cd-hit-est (*68*) with a 95% similarity cutoff. To exclude potential contaminations with food, the screening database was composed of the nucleotide sequences derived from the genome projects of nine species of protists and diatomic algae (see table S3). The assembled transcriptomes were screened against this database using BLASTN with *E* value cutoff of 1 × 10^−20^. BUSCO (*52*) values for the transcriptome assemblies are shown in table S2.

Open reading frames (ORFs) were predicted with TransDecoder v5.5.0 (*69*) with the length of 70 amino acids as a lower cutoff value. Redundant peptide isoforms derived from the same genes of allelic variants and ORFs from breakdown transcripts were removed by cd-hit (*68*) with a cutoff value of 95%. Predicted proteins from the newly sequenced transcriptomes were additionally screened against the reference proteomes of 23 species including protists, diatomic algae, nematodes, cnidarians, and three species of Lophotrochozoa as positive controls (complete list of species used for screening is shown in table S4). We expected that the majority of the sequences truly belonging to the Entoprocta and Bryozoa should have their best BLASTP hit among the representatives of Lophotrochozoa. The peptide sets were screened against the database with a BLASTP cutoff of 1 × 10^−5^. If the best BLASTP hit of a peptide had been assigned to potentially contaminating organism, then this sequence has been excluded from further analysis. To estimate the ratio of false-positive hits, the proteome of *H. robusta* (leech) was used as a positive control representing a genome-derived lophotrochozoan dataset. Our two-step screening approach allowed effective exclusion of the protein sequences from diatom algae, which represent a typical food of the suspension feeders and the proteins of ciliates and hydrozoan cnidarians that are known to grow on the colonies of Ectoprocta and Entoprocta. BLASTP screening results and statistics are shown in table S5.

### Taxonomic sampling

To prepare a comprehensive dataset for the phylogenetic placement of Ectoprocta and Entoprocta, we combined protein sets from the newly sequenced transcriptomes with the datasets, which were published previously (see table S6). Several species representing Cnidaria, Ecdysozoa, and Deuterostomia were used as outgroups since their relative placement in the animal phylogeny, and the topology of branches within each group is well established. Prior knowledge about the relationships of the taxa within outgroups is highly important because it allows us to evaluate the validity of marker selection by checking the topology within the outgroup clades in the resulting trees. Only the genome-derived sequences were used for outgroup species. In the final dataset comprising 37 species (see table S6), we used proteins from the genome projects and from the transcriptomes because for several taxa (for example, Nemertea), only one genome has been sequenced so far. Genomes are the most preferential source of protein datasets, but using just one species with the sequenced genome would make the dataset unbalanced and could increase the probability of artifacts such as long branch attraction. To represent Annelida, we took the proteomes of two species, *Capitella teleta* and *Alitta virens*, which have slower evolution rate than the leach *H. robusta* (*70*). Since the sister relations of Brachiopoda and Phoronida have previously obtained strong support (*15*, *16*, *33*, *71*), we decided to use only the genome-derived proteomes and selected two species of brachiopods (*Lingula anatina* and *L. unguis*), as well as the phoronid (*Phoronis australis*), to represent nonbryozoan lophophorates. For all the remaining taxonomic groups, at least three representative species were used. Transcriptomes of two bryozoan species (*Bugula stolonifera* and *Membranipora membranacea*) were assembled de novo from the available NCBI Sequence Read Archive (SRA) data, and their peptide sequences were predicted and processed as described earlier. A similar approach was applied to NCBI SRA data of two species of Nemertea (*Cerebratulus* sp. and *Malacobdella grossa*). The use of SRA allowed us not only to increase the number of species available for phylogeny reconstruction but also, at the same time, to ensure that identical procedures for assembly, peptide prediction, and quality screening had been applied to all transcriptome-based datasets (see table S6).

### Orthology assignment and the protein sets used

Proteomes from six newly sequenced species were combined with 31 proteomes from NCBI/UniProt (see table S6), and orthologous proteins were identified by OrthoFinder v2.4.0 (*72*) with default settings. Protein sequences and information about their assignments to the OGs were imported into MySQL database and the set of 1059 genes present in all species (referred as set S21) was used to infer phylogenetic relationships (see flowchart in fig. S1). For newly sequenced species, the protein isoforms with the highest read support in RNA sequencing (RNA-seq) were selected in the cases when several alternative protein isoforms per gene were present.

Since it has been shown previously that the placement of Ectoprocta (Bryozoa) is strongly influenced by marker selection (*33*), which may also apply to Entoprocta, we decided to test how partitioning of genes based on their evolutionary rates might affect the topology of the resulting trees. On the basis of the initial set of 1059 OGs, several matrices with varying number of genes were generated. For each OG of the initial set S21, gene trees were calculated by RAxML with LG + G substitution model, and three protein subsets with 916 (set AA21), 515 (set AB21), and 369 (set AC21) genes were selected on the basis of mean BS values, the sum of branch lengths and the proportion of gaps (Fig. 3, A to C, and fig. S1). To remove outliers, increasingly stringent criteria were applied to select the genes. For the sets AA21 and AB21, only the genes with mean BS above 40 and 53.47% (median of BS distribution) were selected, respectively. Set AC21 contains 369 OGs where the sum of branch length is below 37.4 (*Q*_3_ + interquartile range × 1.5), the mean BS is above 53.47% (the median value), and the percentage of gaps is below 16.939 (the third quartile).

Next, proteins belonging to BUSCOs were identified in the set S21 using metazoa_odb9 BUSCO database as a reference (*52*, *73*). By definition, BUSCO proteins are encoded by single-copy genes in the majority of metazoans, making them ideal markers for phylogeny reconstruction. Metazoa_odb9 set contains 978 OGs, each group represented by 10 proteins from different taxa, for a total set of 9780 proteins (/ancestral_variants file at http://busco.ezlab.org/v2/datasets/metazoa_odb9.tar.gz). Proteomes from our dataset and the BUSCO metazoa_odb9 set (9780 proteins) were combined together and assigned to OGs by OrthoFinder v2.4.0 with *mcl* inflation rate *I* = 1.5. Thus, all the proteins belonging to BUSCO OGs were identified for each species (table S7). With the default parameters OrthoFinder placed 9780 BUSCO proteins in 1096 OGs, with 766 groups being represented by 10 members as expected, and 330 OGs had less than 10 BUSCO proteins. Next, we selected those OGs with BUSCO proteins that met two conditions: (i) For every species selected for the analysis, at least one protein must be present; and (ii) all 10 orthologs from the BUSCO metazoa_odb9 set must be assigned to this OG. The resulting set AE21 included 36 taxa (one nemertean species, *M. grossa*, was excluded to increase the total number of available BUSCO genes) and contained proteins from 247 OGs. Annotations and evolutionary rates of the genes included into set AE21 are listed in the table S7.

The set AH21 was derived from the set S21 (1059 genes) by completely removing the alignment columns containing even a single gap among all 37 taxa. Thus, a matrix with 100% completeness and 16,415 amino acid positions in total has been obtained. Of 1059 genes present in set S21, 340 genes (32.1%) were retained in the set AH21.

### Data recoding schemes

To evaluate the potential impact of compositional heterogeneity, the alignment matrices of the sets AE21 and AH21 were recoded into 6, 9, and 15 states based on PAM250 transition matrix. Recoding strategies with 9 and 15 states were shown previously to produce fewer incorrect trees than the Dayhoff-6 recording scheme on simulated data with various levels of compositional heterogeneity (*54*). The following binning schemes were applied: standard Dayhof-6 (*53*) [(DENQ), (ILMV), (FYW), (ASTGP), (HKR), C], 9-state recoding [(DEHNQ), (ILMV), (FY), (AST), (KR), G, P, C, W] referred to as Dayhoff-9, and a 15-state recoding [(DEQ), (ML), (IV), (FY), G, A, P, S, T, N, K, H, R, C, W] referred to as Dayhoff-15. The recoded and nonrecoded versions of the matrices were analyzed in Phylobayes-MPI 1.8c using CAT + GTR model (*74*).

### Phylogenetic analyses

Because of the excellent completeness of the proteomes used, we decided not to allow any missing genes in our data matrices, namely, all orthologs selected for ML and BI analysis must have been present in all species analyzed. Proteins belonging to each OG were aligned with MAFFT v.7.130b (*75*) (with --maxiterate 1000 --localpair --leavegappyregion options) and poorly aligned areas were removed with TrimAL v.1.2rev.59 with -gappyout option (*76*). Alignments of individual genes were concatenated into a supermatrix using FASconCAT-G_v1.02 (*77*). Further analyses were carried out using ML and BI in MPI version of RAxML 8.2.4 (raxmlHPC-MPI-AVX) (*78*) and Phylobayes-MPI 1.8c (*74*), respectively. For ML analyses, 100 rapid bootstrap inferences followed by a thorough ML search were used. Data matrices for ML analyses were partitioned by genes except for the set AH21 where partitioning has not been applied. For BI, two to four chains were run for each data matrix in parallel, and their convergence was tested by monitoring maxdiff and meandiff values calculated by *bpcomp* program. The CAT + GTR + G model was used in all Bayesian analyses. Depending on the matrix from 11,000 to 63,000 trees per chain were calculated. The calculations were stopped when convergence or stable equilibrium state has been reached. After that, the majority rule consensus tree was computed with at least two-thirds of initial points discarded as burn-in. Composition heterogeneity was assessed by PPA performed with -allppred option of *readpb_mpi* program. Chains that reached convergence or equilibrium state were used for PPA, and a *z* score was used to measure the deviation of data from the homogenous compositional distribution. The phylogenetic trees obtained in ML and BI analyses were visualized and rerooted with cnidarian outgroup in FigTree-v1.4 (*79*).

### Coalescence of gene trees and distribution of shared orthologs

The species tree was calculated with ASTRAL-III v5.15.4 (*55*) based on the set of 1036 gene trees derived from the set S21 (Fig. 5A and setS21′ in fig. S1). Number is lower than 1059 because in 23 cases one of the protein sequences in the alignment was trimmed completely in at least one of the species. Individual gene trees were reconstructed by RAxML 8.2.4 with the LG + G model. Since the removal of branches with very low support improves accuracy (*55*), all the branches in the ML trees with a BS of ≤10% were contracted using Newick Utils 1.6 (*80*) (nw_ed 'i & b < =10' option) before consensus tree calculation. Coalescent trees based on 515 gene trees (set AB21) and 369 gene trees (set AC21) are shown in fig. S5 (A and B, respectively). ASTRAL estimates branch lengths for internal branches only, and the branch support values measure the support for a quadripartition (the four clusters around a branch) and not for the bipartition, as is commonly done in other programs.

Recently, several genes potentially important for taxon-specific functions and structures in Lophotrochozoa have been identified (*35*, *81*). The presence of these genes may not only reflect convergent evolution but also indicate common evolutionary paths. Similarities and differences in gene sets among the representatives of Ectoprocta and Entoprocta, as well as their comparison with a wider range of Lophotrochozoa, may provide additional insights about their evolutionary history and phylogenetic placement. To compare gene sets, we identified orthologs in the proteomes of 37 species with OrthoFinder, and the species were clustered using the number of shared OGs as a measure of similarity. Complete correlation option of *pheatmap* R library was used for clustering and visualization. Since the presence of OGs, not the presence of individual genes is compared, this analysis is rather stable against copy-number variations, the presence of paralogs, and multiple protein isoforms that might be present in the RNA-seq–based proteomes.

### Scanning electron microscopy

The material was fixed in 2.5% glutaraldehyde solution in 0.1 M isotonic cacodylate buffer (supplemented with sucrose to reach 750 mosmol), then processed for scanning electron microscopy (SEM) study according to standard protocols, critical point–dried, and sputter-coated with 20-nm gold. Scanning electron micrographs were made using a Tescan MIRA3 LMU (Tescan, Brno, Czech Republic) scanning electron microscope.

## Notes

View/request a protocol for this paper from *Bio-protocol*.

## References

[1] K. M. Halanych, J. D. Bacheller, A. M. Aguinaldo, S. M. Liva, D. M. Hillis, J. A. Lake, Evidence from 18S ribosomal DNA that the lophophorates are protostome animals. Science 267, 1641–1643 (1995). 7886451 10.1126/science.7886451

[2] A. M. Aguinaldo, J. M. Turbeville, L. S. Linford, M. C. Rivera, J. R. Garey, R. A. Raff, J. A. Lake, Evidence for a clade of nematodes, arthropods and other moulting animals. Nature 387, 489–493 (1997). 9168109 10.1038/387489a0

[3] C. W. Dunn, A. Hejnol, D. Q. Matus, K. Pang, W. E. Browne, S. A. Smith, E. Seaver, G. W. Rouse, M. Obst, C. D. Edgecombe, M. V. Sørensen, S. H. D. Haddock, A. Schmidt-Rhaesa, A. Okusu, R. M. Kristensen, W. C. Wheeler, M. Q. Martindale, G. Giribet, Broad phylogenomic sampling improves resolution of the animal tree of life. Nature 452, 745–749 (2008). 18322464 10.1038/nature06614

[4] K. M. Halanych, The new view of animal phylogeny. Annu. Rev. Ecol. Evol. Syst. 35, 229–256 (2004).

[5] D. Q. Matus, R. R. Copley, C. W. Dunn, A. Hejnol, H. Eccleston, K. M. Halanych, M. Q. Martindale, M. J. Telford, Broad taxon and gene sampling indicate that chaetognaths are protostomes. Curr. Biol. 16, R575–R576 (2006). 16890509 10.1016/j.cub.2006.07.017

[6] C. W. Dunn, G. Giribet, G. D. Edgecombe, A. Hejnol, Animal phylogeny and its evolutionary implications. Annu. Rev. Ecol. Evol. Syst. 45, 371–395 (2014).

[7] G. Giribet, Assembling the lophotrochozoan (=spiralian) tree of life. Phil. Trans. R. Soc. B 363, 1513–1522 (2008). 18192183 10.1098/rstb.2007.2241 PMC2614230

[8] G. Giribet, New animal phylogeny: Future challenges for animal phylogeny in the age of phylogenomics. Org. Divers. Evol. 16, 419–426 (2016).

[9] A. Hejnol, M. Obst, A. Stamatakis, M. Ott, G. W. Rouse, G. D. Edgecombe, P. Martinez, J. Baguñà, X. Bailly, U. Jondelius, M. Wiens, W. E. G. Müller, E. Seaver, W. C. Wheeler, M. Q. Martindale, G. Giribet, C. W. Dunn, Assessing the root of bilaterian animals with scalable phylogenomic methods. Proc. R. Soc. B Biol. Sci. 276, 4261–4270 (2009). 10.1098/rspb.2009.0896 PMC2817096 19759036

[10] G. D. Edgecombe, G. Giribet, C. W. Dunn, A. Hejnol, R. M. Kristensen, R. C. Neves, G. W. Rouse, K. Worsaae, M. V. Sørensen, Higher-level metazoan relationships: Recent progress and remaining questions. Org. Divers. Evol. 11, 151–172 (2011).

[11] C. Nielsen, *Animal Evolution. Interrelationships of the Living Phyla* (Oxford Univ. Press, 2012).

[12] T. H. Struck, A. R. Wey-Fabrizius, A. Golombek, L. Hering, A. Weigert, C. Bleidorn, S. Klebow, N. Iakovenko, B. Hausdorf, M. Petersen, P. Kück, H. Herlyn, T. Hankeln, Platyzoan paraphyly based on phylogenomic data supports a noncoelomate ancestry of spiralia. Mol. Biol. Evol. 31, 1833–1849 (2014). 24748651 10.1093/molbev/msu143

[13] K. M. Kocot, On 20 years of Lophotrochozoa. Org. Divers. Evol. 16, 329–343 (2016).

[14] K. M. Kocot, T. H. Struck, J. Merkel, D. S. Waits, C. Todt, P. M. Brannock, D. A. Weese, J. T. Cannon, L. L. Moroz, B. Lieb, K. M. Halanych, Phylogenomics of Lophotrochozoa with consideration of systematic error. Syst. Biol. 66, 256–282 (2017). 27664188 10.1093/sysbio/syw079

[15] F. Marlétaz, K. T. Peijnenburg, T. Goto, N. Satoh, D. S. Rokhsar, A new spiralian phylogeny places the enigmatic arrow worms among Gnathiferans. Curr. Biol. 29, 312–318.e3 (2019). 30639106 10.1016/j.cub.2018.11.042

[16] C. E. Laumer, R. Fernández, S. Lemer, D. Combosch, K. M. Kocot, A. Riesgo, S. C. S. Andrade, W. Sterrer, M. V. Sørensen, G. Giribet, Revisiting metazoan phylogeny with genomic sampling of all phyla. Proc. Biol. Sci. 286, 20190831 (2019). 31288696 10.1098/rspb.2019.0831 PMC6650721

[17] J. V. Thompson, Memoir V. On Polyzoa, a new animal discovered as an inhabitant of some zoophites – with a description of the newly instituted genera of Pedicellaria and Vesicularia, and their Species. Zool. Res. 4, 89–102 (1830).

[18] C. G. Ehrenberg, *Symbolæ Physicæ, Seu icones et Descriptiones Animalium Evertebratorum* (G. Reimer, 1831).

[19] H. Nitsche, Beiträge zur Kenntnis der Bryozoen. Z. wiss. Zool. 20, 1–36 (1869).

[20] B. Hatschek, *Lehrbuch der Zoologie, eine morphologische Ubersicht des Thierreiches zur Einfuhrung in das Studium dieser Wissenschaft. Erste Lieferung* (Jena G. Fisher, 1888).

[21] L. H. Hyman, *The Invertebrates, vol. 5. Smaller Coelomate Groups* (McGraw Hill, 1959).

[22] P. Brien, L. Papyn, Les Endoproctes et la Classe des Bryozoaires. Ann. Soc. R. Zool. Belg. 85, 59–87 (1954).

[23] A. Wanninger, J. Fuchs, G. Haszprunar, Anatomy of the serotonergic nervous system of an entoproct creeping-type larva and its phylogenetic implications. Invertebr. Biol. 126, 268–278 (2007).

[24] G. Haszprunar, A. Wanninger, On the fine structure of the creeping larva of *Loxosomella murmanica*: Additional evidence for a clade of Kamptozoa (Entoprocta) and Mollusca. Acta Zool. 89, 137–148 (2008).

[25] C. Nielsen, Entoproct life-cycles and the entoproct/ectoproct relationship. Ophelia 9, 209–341 (1971).

[26] C. Nielsen, The relationships of Entoprocta, Ectoprocta and Phoronida. Am. Zool. 17, 149–150 (1977).

[27] C. Nielsen, Animal phylogeny in the light of the trochaea theory. Biol. J. Linn. Soc. 25, 243–299 (1985).

[28] C. Nielsen, The phylogenetic position of Entoprocta, Ectoprocta, Phoronida, and Brachiopoda. Integr. Comp. Biol. 42, 685–691 (2002). 21708765 10.1093/icb/42.3.685

[29] P. Funch, R. M. Kristensen, Cycliophora is a new phylum with affinities to Entoprocta and Ectoprocta. Nature 378, 711–714 (1995).

[30] T. Cavalier-Smith, A revised six-kingdom system of life. Biol. Rev. Camb. Philos. Soc. 73, 203–266 (1998). 9809012 10.1017/s0006323198005167

[31] M. P. Nesnidal, M. Helmkampf, I. Bruchhaus, B. Hausdorf, Compositional heterogeneity and phylogenomic inference of metazoan relationships. Mol. Biol. Evol. 27, 2095–2104 (2010). 20382658 10.1093/molbev/msq097

[32] M. P. Nesnidal, M. Helmkampf, A. Meyer, A. Witek, I. Bruchhaus, I. Ebersberger, T. Hankeln, B. Lieb, T. H. Struck, B. Hausdorf, New phylogenomic data support the monophyly of Lophophorata and an Ectoproct–Phoronid clade and indicate that Polyzoa and Kryptrochozoa are caused by systematic bias. BMC Evol. Biol. 3, 253 (2013). 10.1186/1471-2148-13-253 PMC4225663 24238092

[33] Y. J. Luo, M. Kanda, R. Koyanagi, K. Hisata, T. Akiyama, H. Sakamoto, T. Sakamoto, N. Satoh, Nemertean and phoronid genomes reveal lophotrochozoan evolution and the origin of bilaterian heads. Nat. Ecol. Evol. 2, 141–151 (2018). 29203924 10.1038/s41559-017-0389-y

[34] C. Bleidorn, Recent progress in reconstructing lophotrochozoan (spiralian) phylogeny. Org. Divers. Evol. 19, 557–566 (2019).

[35] S. Santagata, Genes with evidence of positive selection as potentially related to coloniality and the evolution of morphological features among the lophophorates and entoprocts. J. Exp. Zool. B Mol. Dev. Evol. 336, 267–280 (2021). 32638536 10.1002/jez.b.22975

[36] J. Zrzavý, S. Mihulka, P. Kepka, A. Bezděk, D. Tietz, Phylogeny of the Metazoa based on morphological and 18S ribosomal DNA evidence. Cladistics 14, 249–285 (1998). 34905826 10.1111/j.1096-0031.1998.tb00338.x

[37] K. J. Peterson, D. J. Eernisse, Animal phylogeny and the ancestry of bilaterians: Inferences from morphology and 18S rDNA gene sequences. Evol. Dev. 3, 170–205 (2001). 11440251 10.1046/j.1525-142x.2001.003003170.x

[38] Y. J. Passamaneck, K. M. Halanych, Evidence from Hox genes that bryozoans are lophotrochozoans. Evol. Dev. 6, 275–281 (2004). 15230967 10.1111/j.1525-142X.2004.04032.x

[39] Y. J. Passamaneck, K. M. Halanych, Lophotrochozoan phylogeny assessed with LSU and SSU data: Evidence of lophophorate polyphyly. Mol. Phylogenet. Evol. 40, 20–28 (2006). 16556507 10.1016/j.ympev.2006.02.001

[40] A. Waeschenbach, M. J. Telford, J. S. Porter, D. T. Littlewood, The complete mitochondrial genome of *Flustrellidra hispida* and the phylogenetic position of Bryozoa among the Metazoa. Mol. Phylogenet. Evol. 40, 195–207 (2006). 16621614 10.1016/j.ympev.2006.03.007

[41] M. Helmkamph, I. Bruchhaus, B. Hausdorf, Phylogenomic analyses of lophophorates (brachiopods, phoronids and bryozoans) confirm the Lophotrochozoa concept. Proc. Biol. Sci. 275, 1927–1933 (2008). 18495619 10.1098/rspb.2008.0372 PMC2593926

[42] B. Hausdorf, M. Helmkampf, A. Meyer, A. Witek, H. Herlyn, I. Bruchhaus, T. Hankeln, T. H. Struck, B. Lieb, Spiralian phylogenomics supports the resurrection of Bryozoa comprising Ectoprocta and Entoprocta. Mol. Biol. Evol. 24, 2723–2729 (2007). 17921486 10.1093/molbev/msm214

[43] B. Hausdorf, M. Helmkamph, M. P. Nesnidal, I. Bruchhaus, Phylogenetic relationships within the lophophorate lineages (Ectoprocta, Brachiopoda and Phoronida). Mol. Phylogenet. Evol. 55, 1121–1127 (2010). 20045074 10.1016/j.ympev.2009.12.022

[44] N. V. Whelan, K. M. Kocot, K. M. Halanych, Employing phylogenomics to resolve the relationships among cnidarians, ctenophores, sponges, placozoans, and bilaterians. Integr. Comp. Biol. 55, 1084–1095 (2015a). 25972566 10.1093/icb/icv037

[45] N. V. Whelan, K. M. Kocot, L. L. Moroz, K. M. Halanych, Error, signal, and the placement of Ctenophora sister to all other animals. Proc. Natl. Acad. Sci. 112, 5773–5778 (2015b). 25902535 10.1073/pnas.1503453112 PMC4426464

[46] R. Feuda, M. Dohrmann, W. Pett, H. Philippe, O. Rota-Stabelli, N. Lartillot, G. Wörheide, G. D. Pisani, Improved modeling of compositional heterogeneity supports sponges as sister to all other animals. Curr. Biol. 27, 3864–3870.e4 (2017). 29199080 10.1016/j.cub.2017.11.008

[47] H. Philippe, D. M. de Vienne, V. Ranwez, B. Roure, D. Baurain, F. Delsuc, Pitfalls in supermatrix phylogenomics. Eur. J. Taxonomy 10.5852/ejt.2017.283 , (2017).

[48] H. Philippe, A. J. Poustka, M. Chiodin, K. J. Hoff, C. Dessimoz, B. Tomiczek, P. H. Schiffer, S. Müller, D. Domman, M. Horn, H. Kuhl, Mitigating anticipated effects of systematic errors supports sister-group relationship between Xenacoelomorpha and Ambulacraria. Curr. Biol. 29, 1818–1826.e6 (2019). 31104936 10.1016/j.cub.2019.04.009

[49] A. K. Redmond, A. McLysaght, Evidence for sponges as sister to all other animals from partitioned phylogenomics with mixture models and recoding. Nat. Commun. 12, 1783 (2021). 33741994 10.1038/s41467-021-22074-7 PMC7979703

[50] B. Roure, D. Baurain, H. Philippe, Impact of missing data on phylogenies inferred from empirical phylogenomic data sets. Mol. Biol. Evol. 30, 197–214 (2013). 22930702 10.1093/molbev/mss208

[51] C. E. Laumer, N. Bekkouche, A. Kerbl, F. Goetz, R. C. Neves, M. V. Sørensen, R. M. Kristensen, A. Hejnol, C. W. Dunn, G. Giribet, K. Worsaae, Spiralian phylogeny informs the evolution of microscopic lineages. Curr. Biol. 25, 2000–2006 (2015). 26212884 10.1016/j.cub.2015.06.068

[52] F. A. Simão, R. M. Waterhouse, P. Ioannidis, E. V. Kriventseva, E. M. Zdobnov, BUSCO: Assessing genome assembly and annotation completeness with single-copy orthologs. Bioinformatics 31, 3210–3212 (2015). 26059717 10.1093/bioinformatics/btv351

[53] M. O. Dayhoff, R. M. Schwartz, B. C. Orcutt, A model of evolutionary change in proteins, in *Atlas of Protein Sequence and Structure*, M. O. Dayhoff, Ed. (Silver Spring (MD): National Biomedical Research Foundation, 1978), vol. 5, suppl. 3, pp. 345–352.

[54] A. M. Hernandez, J. F. Ryan, Six-state amino acid recoding is not an effective strategy to offset compositional heterogeneity and saturation in phylogenetic analyses. Syst. Biol. 70, 1200–1212 (2021). 33837789 10.1093/sysbio/syab027 PMC8513762

[55] C. Zhang, M. Rabiee, E. Sayyari, S. Mirarab, ASTRAL-III: Polynomial time species tree reconstruction from partially resolved gene trees. BMC Bioinformatics 19, 153 (2018). 29745866 10.1186/s12859-018-2129-y PMC5998893

[56] G. Giribet, G. D. Edgecombe, *The Invertebrate Tree of Life* (Princeton Univ. Press, 2020).

[57] P. Funch, The chordoid larva of *Symbion pandora* (Cycliophora) is a modified trochophore. J. Morphol. 230, 231–263 (1996). 29852661 10.1002/(SICI)1097-4687(199612)230:3<231::AID-JMOR1>3.0.CO;2-H

[58] A. Pross, Untersuchungen zur Gliederung von *Lingula anatina* (Brachiopoda)-Archimerie bei Brachiopoden. Zool Jb Anat Ontog Tiere 103, 250–263 (1980).

[59] A. Williams, M. A. James, C. C. Emig, S. Mackay, M. C. Rhodes, Brachiopod anatomy, in *Treatise on Invertebrate Paleontology, Part H: Brachiopoda*, R. L. Kaesler, Ed. (Boulder, Colorado: The Geological Society of America Inc., Lawrence, Kansas: The University of Kansas, 1997), vol. 1, pp 7–189.

[60] T. V. Kuzmina, V. V. Malakhov, The periesophageal celom of the articulate brachiopod *Hemithyris psittacea* (Rhynchonelliformea, Brachiopoda*)*. J. Morphol. 272, 180–190 (2011). 21210489 10.1002/jmor.10904

[61] E. N. Temereva, V. V. Malakhov, Metamorphic remodeling of morphology and the body cavity in Phoronopsis harmeri (Lophotrochozoa, Phoronida): The evolution of the phoronid body plan and life cycle. BMC Evol. Biol. 15, 229 (2015). 26489660 10.1186/s12862-015-0504-0 PMC4618516

[62] N. Shunatova, Y. Tamberg, Body cavities in bryozoans: Functional and phylogenetic implications. J. Morphol. 280, 1332–1358 (2019). 31251428 10.1002/jmor.21034

[63] N. Shunatova, S. Denisova, S. Shchenkov, Ultrastructure of rhizoids in the marine bryozoan Dendrobeania fruticosa (Gymnolaemata: Cheilostomata). J. Morphol. 282, 847–862 (2021). 33759196 10.1002/jmor.21351

[64] E. N. Temereva, E. B. Tsitrin, Modern data on the Innervation of the Lophophore in *Lingula anatina* (Brachiopoda) support the monophyly of the Lophophorates. PLOS ONE 10, e0123040 (2015). 25901745 10.1371/journal.pone.0123040 PMC4406759

[65] E. N. Temereva, Innervation of the lophophore suggests that the phoronid Phoronis ovalis is a link between phoronids and bryozoans. Sci. Rep. 7, 14440 (2017). 29089576 10.1038/s41598-017-14590-8 PMC5663845

[66] A. M. Bolger, M. Lohse, B. Usadel, Trimmomatic: A flexible trimmer for Illumina sequence data. Bioinformatics 30, 2114–2120 (2014). 24695404 10.1093/bioinformatics/btu170 PMC4103590

[67] M. G. Grabherr, B. J. Haas, M. Yassou, J. Z. Levin, D. A. Thompson, I. Amit, X. Adiconis, L. Fan, R. Raychowdhury, Q. Zeng, Z. Chen, E. Mauceli, N. Hacohen, A. Gnirke, N. Rhind, F. di Palma, B. W. Birren, C. Nusbaum, K. Lindblad-Toh, N. Friedman, A. Regev, Full-length transcriptome assembly from RNA-seq data without a reference genome. Nat. Biotechnol. 29, 644–652 (2011). 21572440 10.1038/nbt.1883 PMC3571712

[68] L. Fu, B. Niu, Z. Zhu, S. Wu, W. Li, CD-HIT: Accelerated for clustering the next-generation sequencing data. Bioinformatics 28, 3150–3152 (2012). 23060610 10.1093/bioinformatics/bts565 PMC3516142

[69] TransDecoder (2018); https://github.com/TransDecoder/TransDecoder/releases/tag/TransDecoder-v5.5.0.

[70] O. Simakov, F. Marletaz, S. J. Cho, E. Edsinger-Gonzales, P. Havlak, U. Hellsten, D. H. Kuo, T. Larsson, J. Lv, D. Arendt, R. Savage, K. Osoegawa, P. de Jong, J. Grimwood, J. A. Chapman, H. Shapiro, A. Aerts, R. P. Otillar, A. Y. Terry, J. Boore, I. V. Grigoriev, D. R. Lindberg, E. C. Seaver, D. A. Weisblat, N. H. Putnam, D. S. Rokhsar, Insights into bilaterian evolution from three spiralian genomes. Nature 493, 526–531 (2013). 23254933 10.1038/nature11696 PMC4085046

[71] Y. J. Luo, T. Takeuchi, R. Koyanagi, L. Yamada, M. Kanda, M. Khalturina, M. Fujie, S. Yamasaki, K. Endo, N. Satoh, The Lingula genome provides insights into brachiopod evolution and the origin of phosphate biomineralization. Nat. Commun. 18, 8301 (2015). 10.1038/ncomms9301 PMC4595640 26383154

[72] D. M. Emms, S. Kelly, OrthoFinder: Phylogenetic orthology inference for comparative genomics. Genome Biol. 20, 238 (2019). 31727128 10.1186/s13059-019-1832-y PMC6857279

[73] R. M. Waterhouse, M. Seppey, F. A. Simão, M. Manni, P. Ioannidis, G. Klioutchnikov, E. V. Kriventseva, E. M. Zdobnov, BUSCO applications from quality assessments to gene prediction and phylogenomics. Mol. Biol. Evol. 35, 543–548 (2018). 29220515 10.1093/molbev/msx319 PMC5850278

[74] N. Lartillot, N. Rodrigue, D. Stubbs, J. Richer, PhyloBayes MPI: Phylogenetic reconstruction with infinite mixtures of profiles in a parallel environment. Syst. Biol. 62, 611–615 (2013). 23564032 10.1093/sysbio/syt022

[75] K. Katoh, D. M. Standley, MAFFT multiple sequence alignment software version 7: Improvements in performance and usability. Mol. Biol. Evol. 30, 772–780 (2013). 23329690 10.1093/molbev/mst010 PMC3603318

[76] S. Capella-Gutiérrez, J. M. Silla-Martínez, T. Gabaldón, trimAl: A tool for automated alignment trimming in large-scale phylogenetic analyses. Bioinformatics 25, 1972–1973 (2009). 19505945 10.1093/bioinformatics/btp348 PMC2712344

[77] P. Kück, K. Meusemann, FASconCAT: Convenient handling of data matrices. Mol. Phylogenet. Evol. 56, 1115–1118 (2010). 20416383 10.1016/j.ympev.2010.04.024

[78] A. Stamatakis, RAxML version 8: A tool for phylogenetic analysis and post-analysis of large phylogenies. Bioinformatics 30, 1312–1313 (2014). 24451623 10.1093/bioinformatics/btu033 PMC3998144

[79] FigTree-1.4.4 (2018); http://tree.bio.ed.ac.uk/software/figtree/.

[80] T. Junier, E. M. Zdobnov, The Newick utilities: High-throughput phylogenetic tree processing in the UNIX shell. Bioinformatics 26, 1669–1670 (2010). 20472542 10.1093/bioinformatics/btq243 PMC2887050

[81] L. Wu, L. S. Hiebert, M. Klann, Y. Passamaneck, B. R. Bastin, S. Q. Schneider, M. Q. Martindale, E. C. Seaver, S. A. Maslakova, J. D. Lambert, Genes with spiralian-specific protein motifs are expressed in spiralian ciliary bands. Nat. Commun. 11, 4171 (2020). 32820176 10.1038/s41467-020-17780-7 PMC7441323

