# Supplementary material for: Polyzoa is back: The effect of complete gene sets on the placement of Ectoprocta and Entoprocta
Source: Sci Adv. 2022 Jul 1;8(26):eabo4400. doi: 10.1126/sciadv.abo4400 (PMC10883361; doi:10.1126/sciadv.abo4400)
Supplement: Supplementary file 1 — Figs. S1 to S5 [file sciadv.abo4400_sm.pdf]

Science Advances
AAAS

Supplementary Materials for

Polyzoa is back: The effect of complete gene sets on the placement of Ectoprocta and Entoprocta

Konstantin Khalturin *et al.*

Corresponding author: Konstantin Khalturin, konstantin.khalturin@oist.jp

Sci. Adv. **8**, eabo4400 (2022)
DOI: 10.1126/sciadv.abo4400

The PDF file includes:

Figs. S1 to S5
Legends for tables S1 to S8

Other Supplementary Material for this manuscript includes the following:

Tables S1 to S8

Figure S1

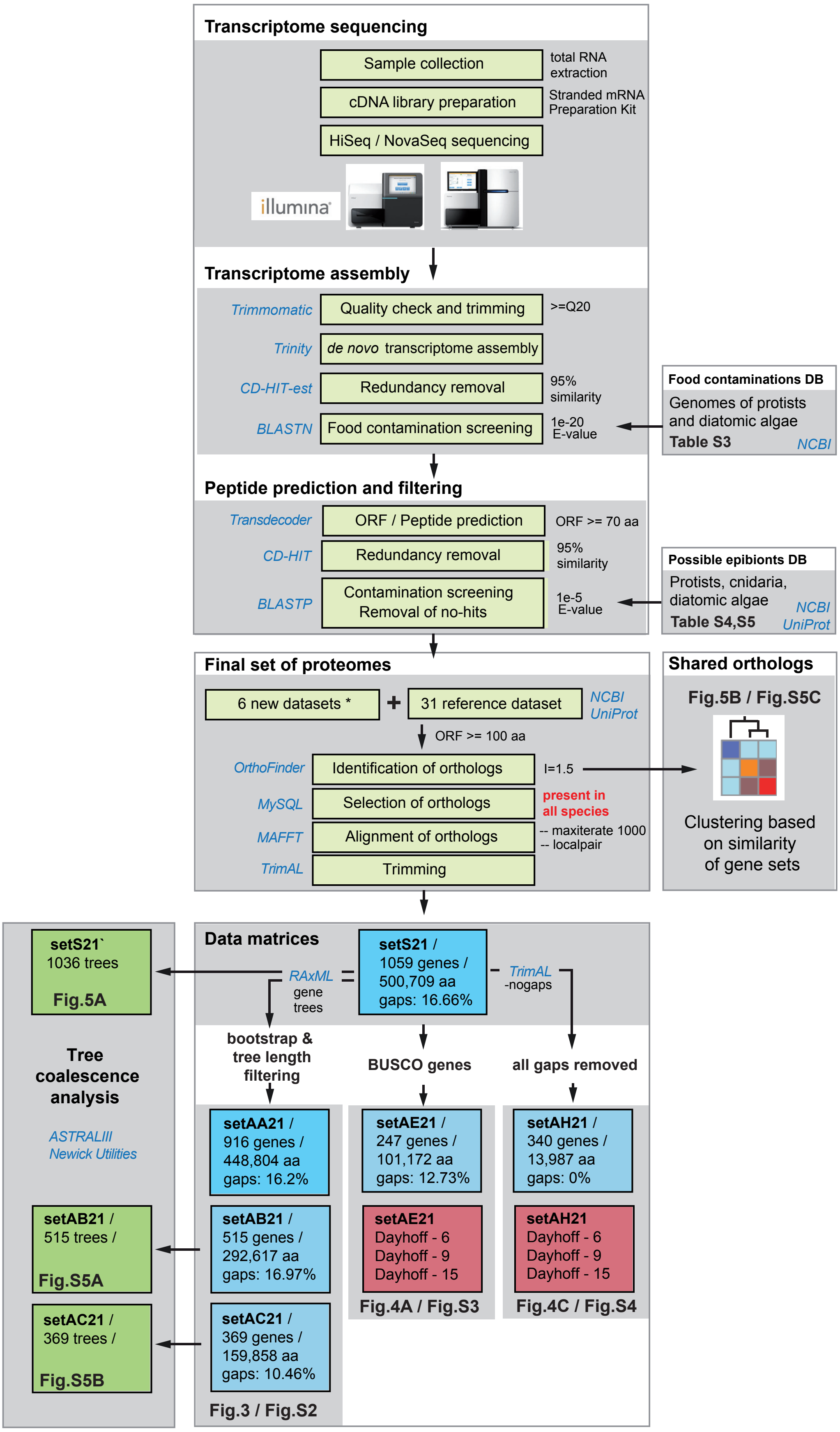

Transcriptome sequencing
Sample collection
total RNA extraction
cDNA library preparation
Stranded mRNA Preparation Kit
HiSeq / NovaSeq sequencing
illumina
Transcriptome assembly
Trimmomatic
Quality check and trimming
>=Q20
Trinity
de novo transcriptome assembly
CD-HIT-est
Redundancy removal
95% similarity
BLASTN
Food contamination screening
1e-20 E-value
Food contaminations DB
Genomes of protists and diatomic algae
Table S3
NCBI
Peptide prediction and filtering
Transdecoder
ORF / Peptide prediction
ORF >= 70 aa
CD-HIT
Redundancy removal
95% similarity
BLASTP
Contamination screening
Removal of no-hits
1e-5 E-value
Possible epibionts DB
Protists, cnidaria, diatomic algae
NCBI
UniProt
Table S4,S5
Final set of proteomes
6 new datasets *
31 reference dataset
NCBI
UniProt
ORF >= 100 aa
OrthoFinder
Identification of orthologs
I=1.5
MySQL
Selection of orthologs
present in all species
MAFFT
Alignment of orthologs
-- maxiterate 1000
-- localpair
TrimAL
Trimming
Shared orthologs
Fig.5B / Fig.S5C
Clustering based on similarity of gene sets
Data matrices
setS21 / 1059 genes / 500,709 aa gaps: 16.66%
RAxML
gene trees
TrimAL -nogaps
setS21` 1036 trees
Fig.5A
Tree coalescence analysis
ASTRALIII
Newick Utilities
bootstrap & tree length filtering
BUSCO genes
all gaps removed
setAA21 / 916 genes / 448,804 aa gaps: 16.2%
setAE21 / 247 genes / 101,172 aa gaps: 12.73%
setAH21 / 340 genes / 13,987 aa gaps: 0%
setAB21 / 515 genes / 292,617 aa gaps: 16.97%
setAE21 Dayhoff - 6 Dayhoff - 9 Dayhoff - 15
setAH21 Dayhoff - 6 Dayhoff - 9 Dayhoff - 15
Fig.4A / Fig.S3
Fig.4C / Fig.S4
setAB21 / 515 trees /
Fig.S5A
setAC21 / 369 genes / 159,858 aa gaps: 10.46%
setAC21 / 369 trees /
Fig.S5B
Fig.3 / Fig.S2

Figure S2

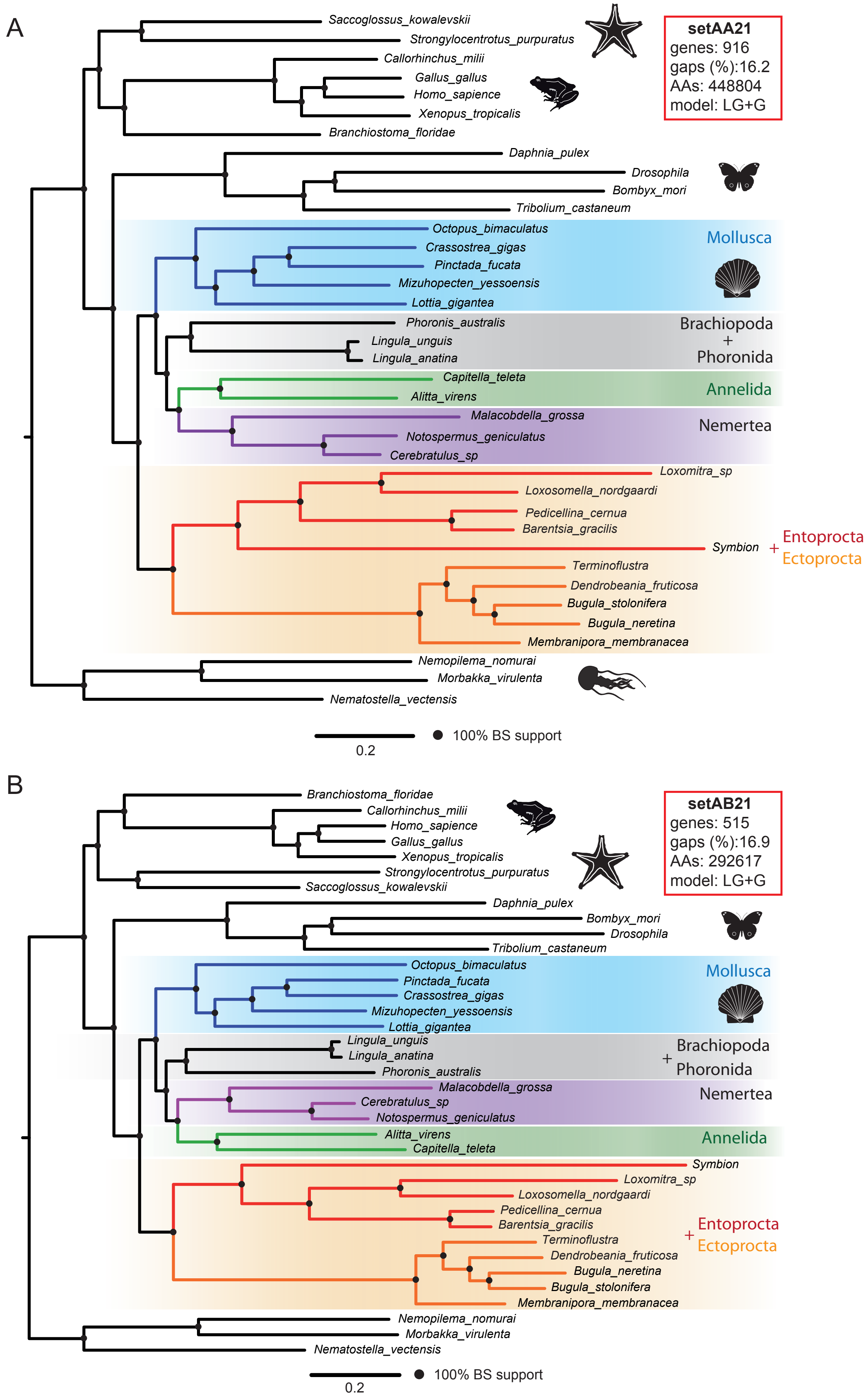
A
Saccoglossus_kowalevskii
Strongylocentrotus_purpuratus
Callorhinchus_milii
Gallus_gallus
Homo_sapience
Xenopus_tropicalis
Branchiostoma_floridae
Daphnia_pulex
Drosophila
Bombyx_mori
Tribolium_castaneum
Octopus_bimaculatus
Crassostrea_gigas
Pinctada_fucata
Mizuhopecten_yessoensis
Lottia_gigantea
Phoronis_australis
Lingula_unguis
Lingula_anatina
Capitella_teleta
Alitta_virens
Malacobdella_grossa
Notospermus_geniculatus
Cerebratulus_sp
Loxomitra_sp
Loxosomella_nordgaardi
Pedicellina_cernua
Barentsia_gracilis
Symbion
Terminoflustra
Dendrobeania_fruticosa
Bugula_stolonifera
Bugula_neretina
Membranipora_membranacea
Nemopilema_nomurai
Morbakka_virulenta
Nematostella_vectensis
setAA21
genes: 916
gaps (%):16.2
AAs: 448804
model: LG+G
Mollusca
Brachiopoda + Phoronida
Annelida
Nemertea
Entoprocta + Ectoprocta
0.2
100% BS support
B
Branchiostoma_floridae
Callorhinchus_milii
Homo_sapience
Gallus_gallus
Xenopus_tropicalis
Strongylocentrotus_purpuratus
Saccoglossus_kowalevskii
Daphnia_pulex
Bombyx_mori
Drosophila
Tribolium_castaneum
Octopus_bimaculatus
Pinctada_fucata
Crassostrea_gigas
Mizuhopecten_yessoensis
Lottia_gigantea
Lingula_unguis
Lingula_anatina
Phoronis_australis
Malacobdella_grossa
Cerebratulus_sp
Notospermus_geniculatus
Alitta_virens
Capitella_teleta
Symbion
Loxomitra_sp
Loxosomella_nordgaardi
Pedicellina_cernua
Barentsia_gracilis
Terminoflustra
Dendrobeania_fruticosa
Bugula_neretina
Bugula_stolonifera
Membranipora_membranacea
Nemopilema_nomurai
Morbakka_virulenta
Nematostella_vectensis
setAB21
genes: 515
gaps (%):16.9
AAs: 292617
model: LG+G
Mollusca
Brachiopoda + Phoronida
Nemertea
Annelida
Entoprocta + Ectoprocta
0.2
100% BS support

Figure S3

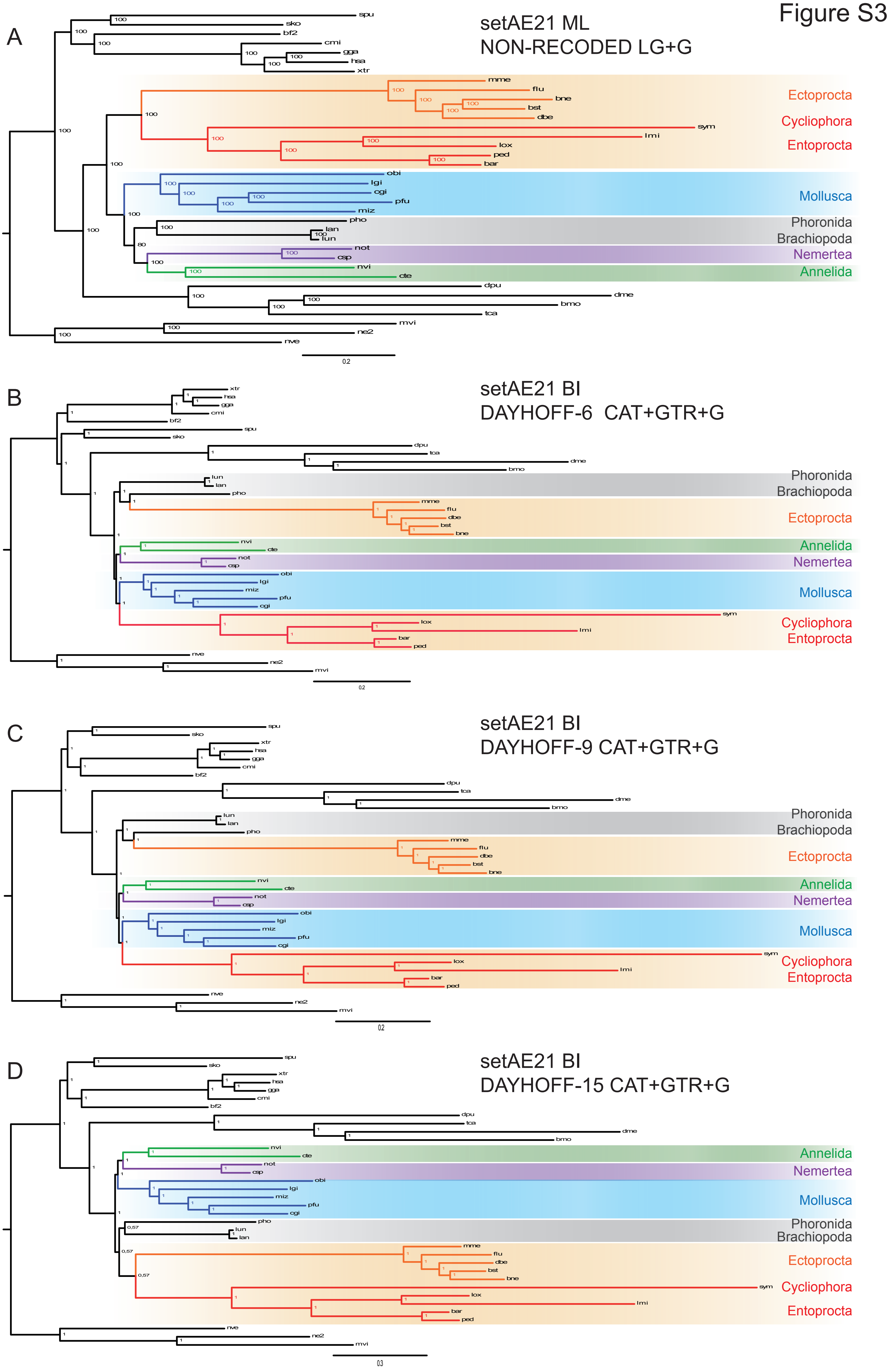

A
setAE21 ML
NON-RECODED LG+G
Ectoprocta
Cycliophora
Entoprocta
Mollusca
Phoronida
Brachiopoda
Nemertea
Annelida
B
setAE21 BI
DAYHOFF-6 CAT+GTR+G
Phoronida
Brachiopoda
Ectoprocta
Annelida
Nemertea
Mollusca
Cycliophora
Entoprocta
C
setAE21 BI
DAYHOFF-9 CAT+GTR+G
Phoronida
Brachiopoda
Ectoprocta
Annelida
Nemertea
Mollusca
Cycliophora
Entoprocta
D
setAE21 BI
DAYHOFF-15 CAT+GTR+G
Annelida
Nemertea
Mollusca
Phoronida
Brachiopoda
Ectoprocta
Cycliophora
Entoprocta

Figure S4

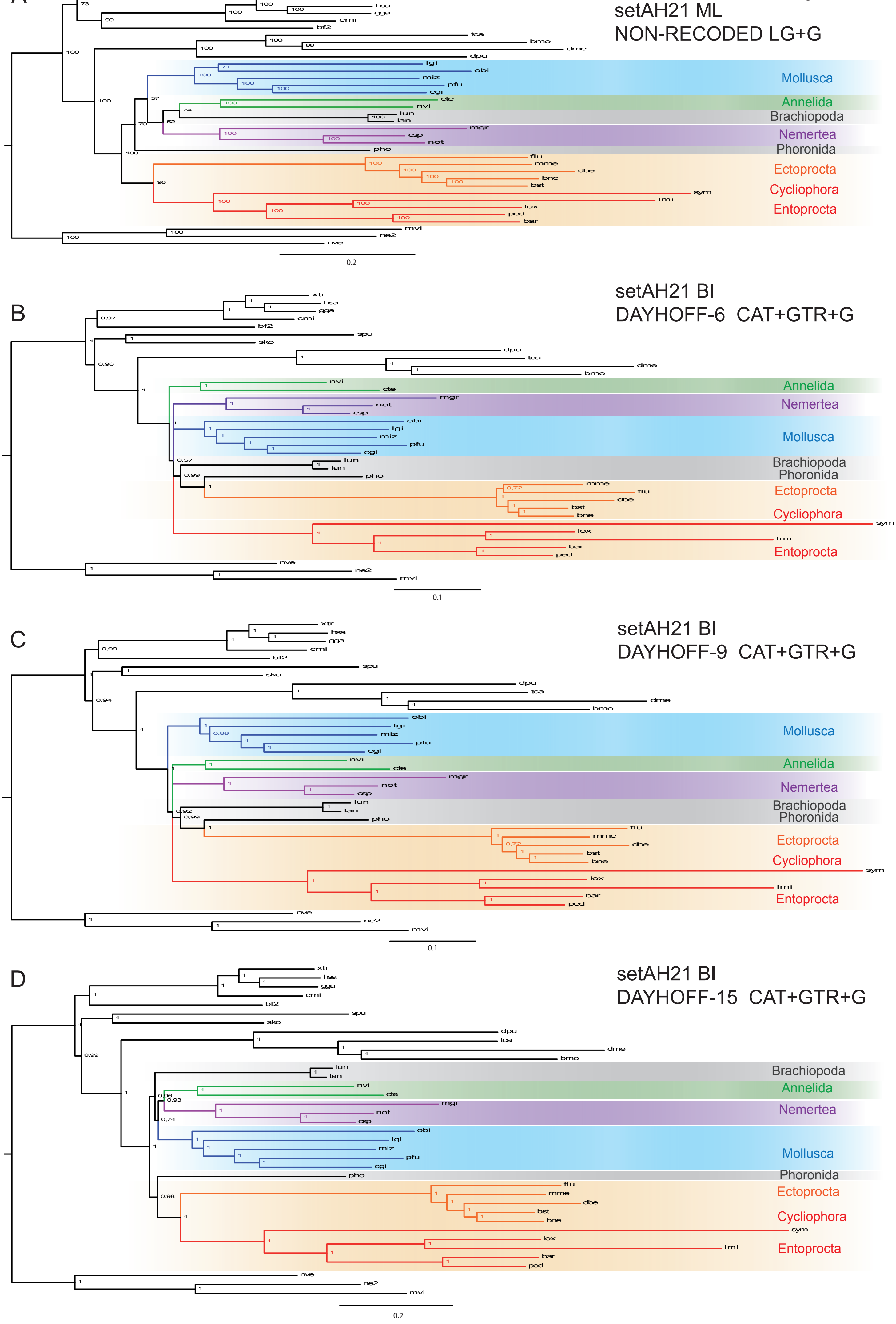
A
setAH21 ML
NON-RECODED LG+G
Mollusca
Annelida
Brachiopoda
Nemertea
Phoronida
Ectoprocta
Cycliophora
Entoprocta
0.2
B
setAH21 BI
DAYHOFF-6 CAT+GTR+G
Annelida
Nemertea
Mollusca
Brachiopoda
Phoronida
Ectoprocta
Cycliophora
Entoprocta
0.1
C
setAH21 BI
DAYHOFF-9 CAT+GTR+G
Mollusca
Annelida
Nemertea
Brachiopoda
Phoronida
Ectoprocta
Cycliophora
Entoprocta
0.1
D
setAH21 BI
DAYHOFF-15 CAT+GTR+G
Brachiopoda
Annelida
Nemertea
Mollusca
Phoronida
Ectoprocta
Cycliophora
Entoprocta
0.2

Figure S5

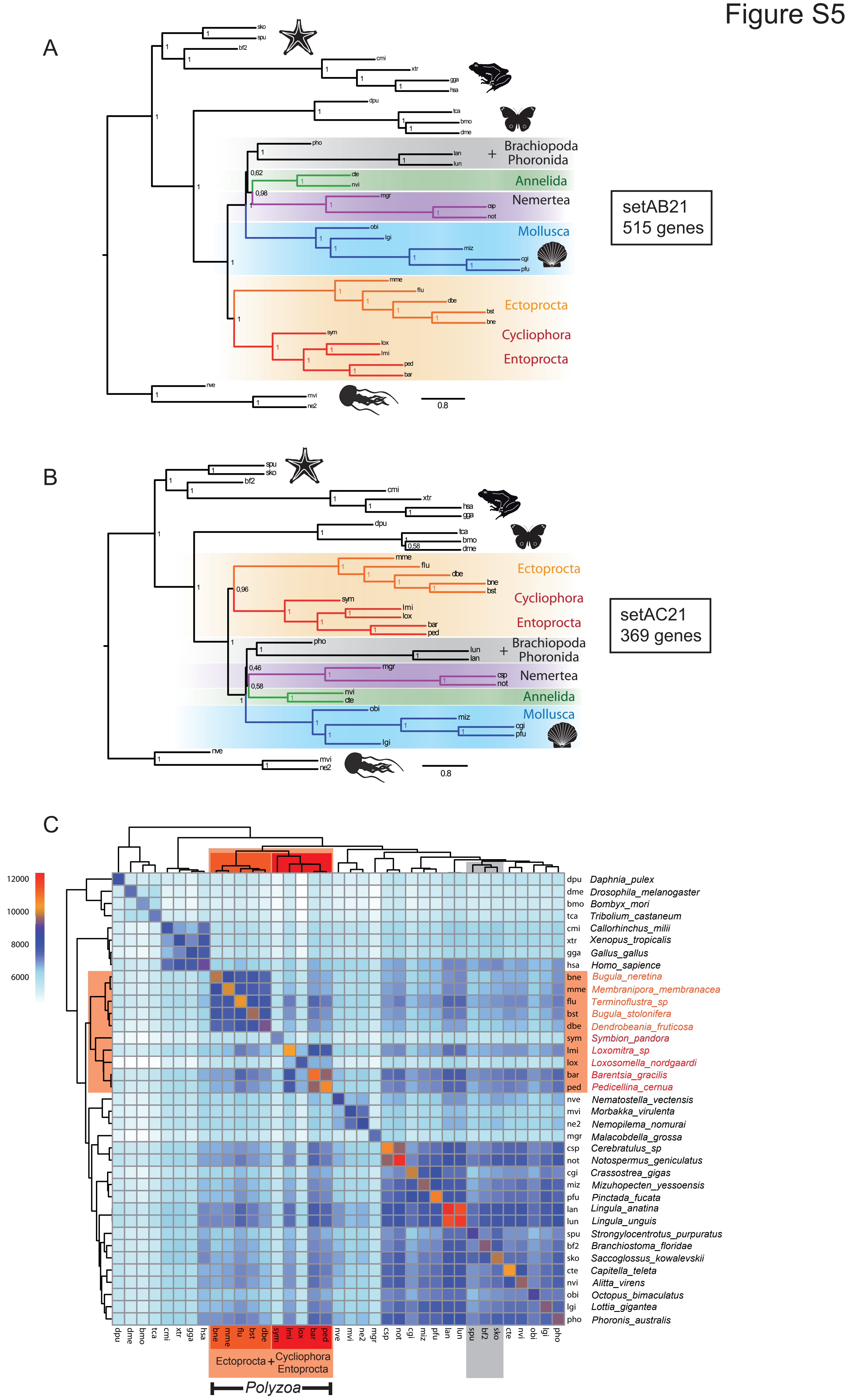
A
setAB21
515 genes
Brachiopoda
Phoronida
Annelida
Nemertea
Mollusca
Ectoprocta
Cycliophora
Entoprocta
0.8
B
setAC21
369 genes
Ectoprocta
Cycliophora
Entoprocta
Brachiopoda
Phoronida
Nemertea
Annelida
Mollusca
0.8
C
12000
10000
8000
6000
dpu Daphnia_pulex
dme Drosophila_melanogaster
bmo Bombyx_mori
tca Tribolium_castaneum
cmi Callorhinchus_milii
xtr Xenopus_tropicalis
gga Gallus_gallus
hsa Homo_sapience
bne Bugula_neretina
mme Membranipora_membranacea
flu Terminoflustra_sp
bst Bugula_stolonifera
dbe Dendrobeania_fruticosa
sym Symbion_pandora
lmi Loxomitra_sp
lox Loxosomella_nordgaardi
bar Barentsia_gracilis
ped Pedicellina_cernua
nve Nematostella_vectensis
mvi Morbakka_virulenta
ne2 Nemopilema_nomurai
mgr Malacobdella_grossa
csp Cerebratulus_sp
not Notospermus_geniculatus
cgi Crassostrea_gigas
miz Mizuhopecten_yessoensis
pfu Pinctada_fucata
lan Lingula_anatina
lun Lingula_unguis
spu Strongylocentrotus_purpuratus
bf2 Branchiostoma_floridae
sko Saccoglossus_kowalevskii
cte Capitella_teleta
nvi Alitta_virens
obi Octopus_bimaculatus
lgi Lottia_gigantea
pho Phoronis_australis
Ectoprocta + Cycliophora Entoprocta
Polyzoa

Supplementary information

Supplementary Figures

Figure S1: A flow chart of the analysis, from sample collection to the resulting data matrices. References to the figures and tables are given. Non-recoded and recoded matrices are shown in blue and red color, respectively.

Figure S2: (A) Maximum likelihood tree based on concatenation of 916 protein markers (set AA21). LG+G substitution model, 448804 distinct alignment patterns, 16.2% of gaps, all branches have maximum bootstrap support. (B) Maximum likelihood tree based on concatenation of 515 protein markers (set AB21). LG+G substitution model, 292617 distinct alignment patterns, 16.9% of gaps, all branches have maximum bootstrap support.

Figure S3: (A) Maximum likelihood tree based on concatenation of 247 protein markers (set AE21). LG+G substitution model, 101172 distinct alignment patterns, 12.7% of gaps, bootstrap support is shown at all internodes. (B-D) Bayesian inference based on concatenation of 247 protein markers from BUSCO set (set AE21). Majority rule consensus trees derived from Dayhoff-6 (B), Dayhoff-9 (C) and Dayhoff-15 (D) recoded matrixes are shown. CAT+GTR+G substitution model, 101172 distinct alignment positions, 12.7% of gaps and undetermined characters, all branches in (B) and (C) have maximum support, in (D) relative positions of Phoronida+Brachiopoda, Ectoprocta and Entoprocta clades are not resolved.

Figure S4: (A) Maximum likelihood tree based on concatenation of 340 protein markers (set AH21). LG+G substitution model, 13987 distinct alignment patterns, 0% of gaps, bootstrap support is shown at all internodes. All nodes except one have maximum support. (B-D) Bayesian inference based on concatenation of 340 protein markers from set AH21. Majority rule consensus trees derived from Dayhoff-6 (B), Dayhoff-9 (C) and Dayhoff-15 (D) recoded matrixes are shown. CAT+GTR+G substitution model in all cases. Posterior probabilities are shown at respective bipartitions.

Figure S5: The consensus trees of coalescence analysis based on 515 orthologs from set AB21 (A) and 369 orthologs from set AC21 (B). All genes were present in all 37 species. Local posterior probabilities are shown at respective bipartitions. Branches with bootstrap support <=10% were collapsed in individual gene trees prior to consensus tree calculation. (C) The heatmap represents the number of orthologous groups (OGs) that are shared among the representatives of all 37 species. The number of shared OGs is coded with a color gradient bar shown on the left side of the figure. Species were clustered based on the similarities of their gene sets. Result of the clustering is represented by a cladogram on the top of the heatmap. Representatives of Ectoprocta, Entoprocta and *Symbion*

(Cycliophora) form a distinct clade. This group of taxa in marked collectively with orange and red polygons as well as with "Polyzoa" tag. The 3-letter species codes are given in addition to species names shown on the right side of the figure. Species names of Ectoporocta and Entoprocta+Cycliophora are shown in orange and red, respectively.

Supplementary Tables

Table S1: Sampling locations and BioProject accessions.

Table S2: Summary of transcriptome assemblies, corresponding RNA-seq libraries and protein sets.

Table S3: Genomes used for food contamination screening (9 genomes - blastn 1e-20).

Table S4: Proteomes used for contamination screening.

Table S5: Screening for potential contaminations (distribution of the best BLASTP hits with E-value cut-off < 1e-5).

Table S6: Proteomes used for phylogeny reconstruction.

Table S7: List of 247 BUSCO genes from set AE21 with corresponding Gene Onthology (GO) and InterProScan annotations (related to Fig.4A-B & Fig.S3).

Table S8: Number of shared orthologous groups among 37 protein sets used in phylogeny reconstruction (Fig.5B & Fig.S5C).
